# Initial Molecular Mechanisms of the Pathogenesis of Parkinson’s Disease in a Mouse Neurotoxic Model of the Earliest Preclinical Stage of This Disease

**DOI:** 10.3390/ijms25021354

**Published:** 2024-01-22

**Authors:** Anna Kolacheva, Ekaterina Pavlova, Alyona Bannikova, Vsevolod Bogdanov, Michael Ugrumov

**Affiliations:** Laboratory of Neural and Neuroendocrine Regulations, Koltzov Institute of Developmental Biology of the Russian Academy of Sciences, 119334 Moscow, Russia; annakolacheva@gmail.com (A.K.); guchia@gmail.com (E.P.); alyona8annikova@yandex.ru (A.B.); vse-bogd@yandex.ru (V.B.)

**Keywords:** Parkinson’s disease, nigrostriatal system, dopaminergic neuron, substantia nigra, striatum, dopamine, tyrosine hydroxylase, 1-methyl-4-phenyl-1,2,3,6-tetrahydropyridine, preclinical model

## Abstract

Studying the initial molecular mechanisms of the pathogenesis of Parkinson’s disease (PD), primarily in the nigrostriatal dopaminergic system, is one of the priorities in neurology. Of particular interest is elucidating these mechanisms in the preclinical stage of PD, which lasts decades before diagnosis and is therefore not available for study in patients. Therefore, our main goal was to study the initial molecular mechanisms of the pathogenesis of PD in the striatum, the key center for dopamine regulation in motor function, in a mouse model of the earliest preclinical stage of PD, from 1 to 24 h after the administration of 1-methyl-4-phenyl-1,2,3,6-tetrahydropyridine (MPTP). It was shown that the content of tyrosine hydroxylase (TH), the first enzyme in dopamine synthesis, does not change within 6 h after the administration of MPTP, but decreases after 24 h. In turn, TH activity increases after 1 h, decreases after 3 h, remains at the control level after 6 h, and decreases 24 h after the administration of MPTP. The concentration of dopamine in the striatum gradually decreases after MPTP administration, despite a decrease in its degradation. The identified initial molecular mechanisms of PD pathogenesis are considered as potential targets for the development of preventive neuroprotective treatment.

## 1. Introduction

Parkinson’s disease (PD) is one of the most severe socially significant neurodegenerative diseases, which can be triggered by either exogenous neurotoxins, such as pesticides, heavy metals, and side products of synthetic heroin [1,2], or endogenous neurotoxins, mainly oligomeric α-synuclein, and the oxidation product of dopamine (DA) [3,4]. PD develops over up to 30 years, in the so-called preclinical stage—without the manifestation of parkinsonian motor symptoms, which are used to diagnose this disease [5,6,7,8]. Over such a long period, endogenous and exogenous neurotoxins cause the death of about half of the nigral dopaminergic (DAergic) neurons, which are a key link in the regulation of motor function [1,3,5,7,9,10]. It is believed that a long course of PD without clinical manifestations is a result of the development of compensatory processes or, in other words, high neuroplasticity.

The above-mentioned characteristics regarding the pathogenesis of PD explain why the current symptomatic treatment of PD patients with DA agonists and l-3,4-dihydroxyphenylalanine (L-DOPA), an immediate precursor to DA, is unable to stop the progression of the disease and avoid disability in patients [8,11,12]. Therefore, one of the highest priorities of neurology is the development of techniques that allow the early (preclinical) diagnosis of PD. Significant progress has already been made in this area of research [13]. Solving this problem will promote the development of preventive neuroprotective therapy aimed at stopping or, at least, slowing down the death of neurons, including nigral DAergic neurons, as well as controlling compensatory processes. Thanks to this, neuroprotective therapy will significantly prolong the preclinical (asymptomatic) stage of the disease, and therefore, the period of normal social and physical activity of potential patients [13].

Since it is not yet possible to diagnose PD in patients at the preclinical stage, preclinical diagnostics and neuroprotective treatments can currently be developed mainly in animal models of the preclinical stage of PD. However, these developments must be preceded by a thorough study of the molecular mechanisms of PD pathogenesis using these models, particularly the degeneration of nigrostriatal DA-producing neurons. There are three fundamentally different types of PD models: genetic, neurotoxic, and combined, but only some neurotoxic models can simulate the progressive stage-by-stage development of PD.

The most effective way to model PD is to use 1-methyl-4-phenyl-1,2,3,6-tetrahydropyridine (MPTP), a precursor to the neurotoxin of DAergic neurons. MPTP is a byproduct in the synthesis of synthetic heroin, causing parkinsonism in drug addicts [2,14]. In the above composition, MPTP use leads to a dose-dependent development of PD—first at the preclinical stage without impairment of motor function, and then at the clinical stage with motor impairment [15]. Based on this, we conducted and published a series of studies on mouse models of acute and subchronic PD at the preclinical stage, shortly before its transition to the clinical stage [16,17]. However, there are still no studies on the initial molecular mechanisms of the pathogenesis of PD using a model of the earliest preclinical stage reproduced by MPTP injection. An analysis of the effect of MPTP on the nigrostriatal dopaminergic system shortly after its administration was carried out in previous studies, but using fundamentally different MPTP administration regimens (either a four-time administration at a dose of 12–20 mg/kg or a single administration at a dose of 30–50 mg/kg), which led to the modeling of the clinical stage of PD [17,18,19,20].

Proceeding from the above, the aim of this work was to study the initial molecular mechanisms of the pathogenesis of PD focusing on the nigrostriatal DAergic system in mice in a model of the earliest preclinical stage of PD.

## 2. Results

### 2.1. The Motor Behavior of Mice

**Figure 1:**
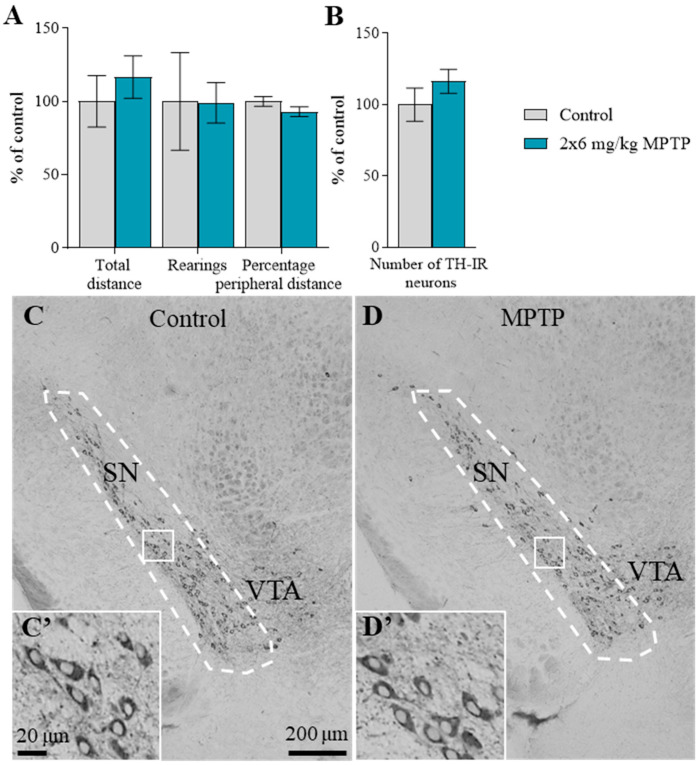
The motor behavior (**A**): the total distance traveled, the number of rearings, and the ratio (in %) of the distance traveled along the periphery of an open field arena to the total distance covered (“*n*” per group = 8); as well as the number of tyrosine hydroxylase-immunopositive neurons in the substantia nigra (SN) (**B**,**C**,**C′**,**D**,**D′**) (“*n*” per group = 3) did not change in mice 24 h after 1-methyl-4-phenyl-1,2,3,6-tetrahydropyridine (MPTP), which was administered twice at a single dose of 6 mg/kg. The white dotted line indicates the SN zone on C and D. VTA—ventral tegmental area. White box—an enlarged fragments from (**C**,**D**) is represented on (**C′**,**D′**). The data in (**A**,**B**) are presented as mean ± SEM, with control taken as 100%. Significant differences with control groups were assessed using the parametric Student’s test (**A**) and the non-parametric Mann–Whitney test (**B**). (**C′**,**D′**) Enlarged fragments from (**C**,**D**), respectively. Bar at (**C**,**D**): 200 μm, (**C′**,**D′**): 20 μm.

The motor behavior did not change in mice 24 h after the administration of MPTP twice at a dose of 6 mg/kg (hereinafter referred to as MPTP administration). There were no changes in any of the assessed parameters: the distance traveled (control: 615 ± 108 cm, MPTP: 716 ± 89 cm), the number of rearings (control: 12 ± 4 units, MPTP: 12 ± 1.7 units), and the ratio of the distance traveled along the periphery of an open field arena to the total distance covered; control: 100 ± 3.28%, MPTP: 93.0 ± 3.29%), compared with the control (Figure 1A).

### 2.2. The Number of Tyrosine Hydroxylase-Immunopositive Neurons in the Substantia Nigra

The number of cell bodies of tyrosine hydroxylase (TH)-immunopositive neurons in the SN in the control is 2283 ± 31.9 (100 ± 1.4%). After 24 h following MPTP administration, the number of such neurons did not change compared with the control, amounting to 2656 ± 192.8 (116 ± 8.5%) (Figure 1B–D).

### 2.3. Nerve Fibers Immunopositive for Tyrosine Hydroxylase, Aromatic L-Amino Acid Decarboxylase, and the Dopamine Transporter in the Striatum of Mice

**Figure 2:**
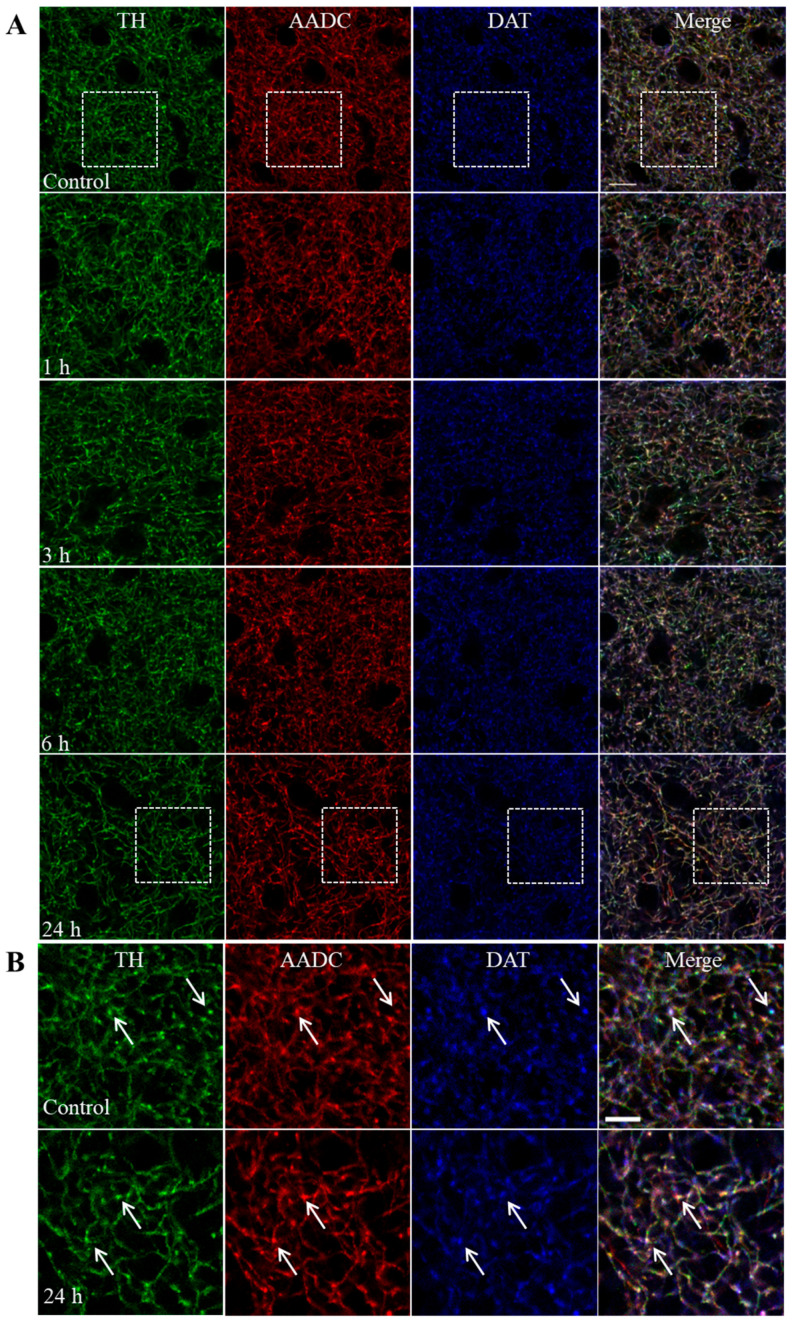
Nerve fibers immunopositive for tyrosine hydroxylase (TH, green), aromatic L-amino acid decarboxylase (AADC, red), and dopamine transporter (DAT, blue) in the striatum of mice 1, 3, 6, and 24 h after double administration of MPTP at a single dose of 6 mg/kg and in the control (**A**). White box—an enlarged fragments from (**A**) (control and 24 h after MPTP injections) is represented on (**B**). Arrows are images of the same nerve fiber after triple labeling. Bar at (**A**): 20 μm, (**B**): 5 μm.

Nerve fibers containing TH, aromatic L-amino acid decarboxylase (AADC), and dopamine transporter (DAT) are shown in Figure 2A,B in the striatum of mice 1, 3, 6 and 24 h after the administration of MPTP and in the control.

**Figure 3:**
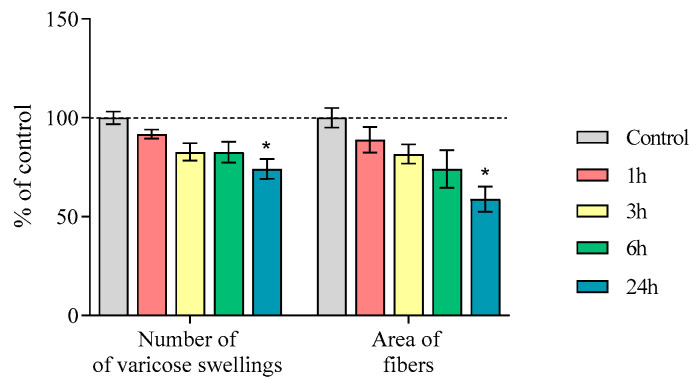
Number of varicose swellings of nerve fibers and area of the nerve fibers immunopositive for tyrosine hydroxylase (TH), aromatic L-amino acid decarboxylase (AADC) and dopamine transporter (DAT) with a diameter 0.3–7 μm^2^ in the striatum (100 μm^2^) of mice (“*n*” per group = 3) 1, 3, 6, and 24 h after double administration of MPTP at a single dose of 6 mg/kg and in controls. Data are presented as mean ± SEM. Control is taken as 100%. * *p* < 0.05 compared with the control group using the nonparametric Mann–Whitney test. Significant differences between groups were assessed using the nonparametric Kruskal–Wallis test.

In Section 2.3, we determined the number of varicose swellings and area of nerve fibers with a diameter of 0.3 to 7 μm^2^ containing TH, AADC, and DAT in the striatum of mice 1, 3, 6, and 24 h after MPTP administration. The number of varicosities per 100 μm^2^ in the control groups was 985.6 ± 31.2, 933.8 ± 12.5, 1089.0 ± 51.3, and 1057.2 ± 50.7, respectively. The area (μm^2^) of the fibers per 100 μm^2^ in the control groups was 970.0 ± 47.8, 870.3 ± 26.9, 1178.6 ± 39.3, and 1172.9 ± 381.9, respectively (Figure 3). A significant reduction in the number of varicose swellings and fiber area was shown after 24 h following MPTP injection, amounting to 74% and 59%, respectively.

### 2.4. The Content of Dopamine, Its Metabolites, and MPP^+^ in the Substantia Nigra

The content (pmol) of DA in the SN in the control groups was as follows: at 1 h—4.07 ± 0.57, at 3 h—4.81 ± 0.31, at 6 h—4.46 ± 0.25, and at 24 h—4.65 ± 0.28. The DOPAC levels were as follows: at 1 h—1.81 ± 0.20, at 3 h—1.93 ± 0.15, at 6 h—2.16 ± 0.12, and at 24 h—1.94 ± 0.20. The HVA levels were as follows: at 1 h—2.00 ± 0.24, at 3 h—2.34 ± 0.11, at 6 h—2.03 ± 0.08, and at 24 h—1.94 ± 0.14 (Table S2).

**Figure 4:**
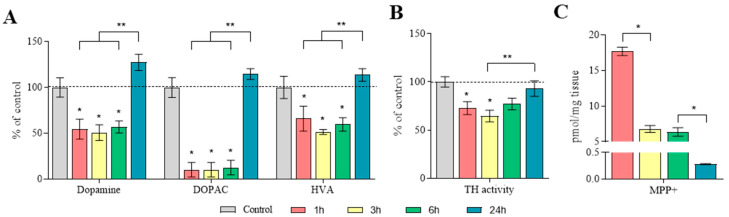
The main characteristics of the substantia nigra as a source of dopamine and a target for 1-methyl-4-phenyl-1,2,3,6-tetrahydropyridine (MPTP), 1 h after saline injections (control) and 1, 3, 6, and 24 h after twice administration of MPTP at a single dose of 6 mg/kg: (**A**) content of dopamine, 3,4-dihydroxyphenylacetic acid (DOPAC), and homovanillic acid (HVA), (“*n*” per group = 8); (**B**) tyrosine hydroxylase (TH) activity, calculated as the difference in the l-3,4-dihydroxyphenylalanine (L-DOPA) content with and without inhibition of aromatic L-amino acid decarboxylase, using 3-hydroxybenzylhydrazine (NSD-1015) (“*n*” per group = 8); and (**C**) concentration of 1-methyl-4-phenylpyridinium (MPP^+^) (“*n*” per group = 8). The data on the content of DA and its metabolites are presented as mean ± SEM, taken as 100% in the control. * *p* < 0.05 compared with the control group (**A**,**B**) or compared with the previous period using the parametric Student’s test (**C**), ** *p* < 0.05 between the selected groups using a one-way analysis of variance with Tukey’s test for post-analysis (**A**,**B**).

At 1, 3, and 6 h after MPTP administration, the DA content in the SN was reduced by 45–50%, the 3,4-dihydroxyphenylacetic acid (DOPAC) content was reduced by 90%, and the homovanillic acid (HVA) content was reduced by 40–50%, relative to the control. After 24 h, the DA, DOPAC, and HVA content increased to the control level (Figure 4A, Table S2). Significant differences in the determination of DA, DOPAC, and HVA concentrations were shown between the samples, obtained 1–6 h and 24 h after MPTP administration.

TH activity in the SN, calculated as the difference between L-DOPA content with and without aromatic L-amino acid decarboxylase (AADC) inhibition by 3-hydroxybenzylhydrazine (NSD-1015), was reduced by 28–36% at 1 and 3 h after MPTP administration (Figure 4B, Table S2). At 6 and 24 h, TH activity in the SN was at the control level (Figure 4B, Table S2). Significant differences were found between the group collected 3 h and 24 h after MPTP administration.

The MPP^+^ concentrations in the SN at 1, 3, 6, and 24 h after MPTP administration were 17.7 ± 0.6, 6.8 ± 0.5, 6.4 ± 0.6, and 0.3 ± 0.01 pmol/mg tissue, respectively (Figure 4C).

**Figure 5:**
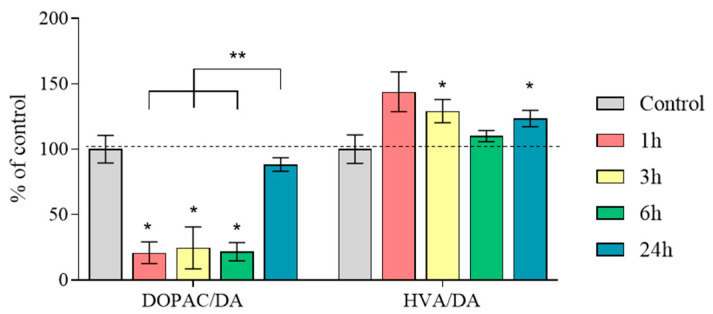
Dopamine (DA) turnover as a ratio of 3,4-dihydroxyphenylacetic acid/DA (DOPAC/DA) and homovanillic acid/DA (HVA/DA) (“*n*” per group = 8) in the substantia nigra 1 h after saline injections (control) and 1, 3, 6, and 24 h after twice administration of 1-methyl-4-phenyl-1,2,3,6-tetrahydropyridine (MPTP) at a single dose of 6 mg/kg. Data are presented as mean ± SEM, taken as 100% in the control. * *p* < 0.05 relative to the control group using the parametric Student’s test, ** *p* < 0.05 between selected groups using one-way analysis of variance with Tukey’s test for post-analysis.

DA turnover, calculated as the DOPAC/DA ratio, was 21%, 25%, and 22% at 1, 3, and 6 h after MPTP administration, respectively (Figure 5, Table S2). After 24 h, this indicator was at the control level (Figure 5, Table S2). Significant differences were found between the group collected 24 h after MPTP administration and all other experimental groups.

DA turnover, calculated as the HVA/DA ratio, was at the control level 1 and 6 h after the administration of MPTP; after 3 and 24 h, it was 129% and 124%, respectively (Figure 5, Table S2).

### 2.5. Concentration of Dopamine, Its Metabolites and MPP^+^, as Well as the Amount of Dopamine Turnover in the Striatum

The concentration (pmol/mg tissue) of DA in the striatum of the control groups was 1 h—103.1 ± 0.9, 3 h—103.9 ± 1.2, 6 h—103.5 ± 1.3, and 24 h—104.7 ± 1.5 (Table S2).

**Figure 6:**
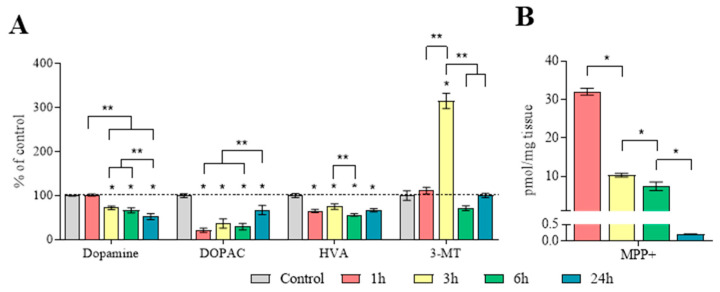
The main characteristics of the striatum as a dopamine collector and target for 1-methyl-4-phenyl-1,2,3,6-tetrahydropyridine (MPTP), 1 h after saline injections (control) and 1, 3, 6, and 24 h after twice administration of MPTP at a single dose of 6 mg/kg: (**A**) concentration of dopamine, 3,4-dihydroxyphenylacetic acid (DOPAC), homovanillic acid (HVA), and 3-methoxytyramine (3-MT) (“*n*” per group = 8); (**B**) concentration of 1-methyl-4-phenylpyridinium (MPP^+^) (“*n*” per group = 8). Data on the concentration of dopamine and its metabolites are presented as mean ± SEM, taken as 100% in the control. * *p* < 0.05 relative to the control group (**A**) or compared with the previous period (**B**) using the parametric Student’s test, ** *p* < 0.05 between the selected groups (**A**) using a one-way analysis of variance with Tukey’s test for post-analysis.

The DA concentration in the striatum 1 h after MPTP administration was at the control level; after 3 and 6 h, it was 67–73%, and by 24 h, it was 52% of the control, taken as 100% (Figure 6A, Table S2). Significant differences were shown between the groups collected 3 and 6 h after MPTP administration and the groups collected 1 and 24 h after MPTP administration, respectively.

The concentration (pmol/mg tissue) of DOPAC in the striatum in the control groups was as follows: at 1 h—7.29 ± 0.30, at 3 h—7.48 ± 0.20, at 6 h—7.15 ± 0.23, and at 24 h—7.35 ± 0.38 (Table S2). Significant differences were shown between the group collected 24 h after MPTP administration and all the other groups.

The concentration (pmol/mg tissue) of HVA in the striatum in the control groups was as follows: at 1 h—9.88 ± 0.46, at 3 h—10.73 ± 0.31, at 6 h—9.80 ± 0.45, and at 24 h—9.59 ± 0.43 (Table S2). The HVA concentration was reduced at 1, 3, 6, and 24 h after MPTP administration by 35%, 25%, 44%, and 33%, respectively (Figure 6A, Table S2). However, significant differences were noted only between the samples obtained 3 and 6 h after MPTP administration.

The concentration (pmol/mg tissue) of 3-methoxytyramine (3-MT) in the striatum in the control groups was as follows: at 1 h—1.28 ± 0.14, at 3 h—1.21 ± 0.11, at 6 h—1.37 ± 0.16, and at 24 h—1.59 ± 0.04 (Table S2). The 3-MT concentration was at the control level 1 h after MPTP administration, while after 3 h, the 3-MT concentration was 314%, and after 6 h and 24 h, this parameter was at the control level (Figure 6A, Table S2).

The MPP^+^ concentrations at 1, 3, 6, and 24 h after MPTP administration were 32.0 ± 0.9, 10.3 ± 0.5, 7.4 ± 1.1, and 0.2 ± 0.0 pmol/mg, respectively (Figure 6B).

**Figure 7:**
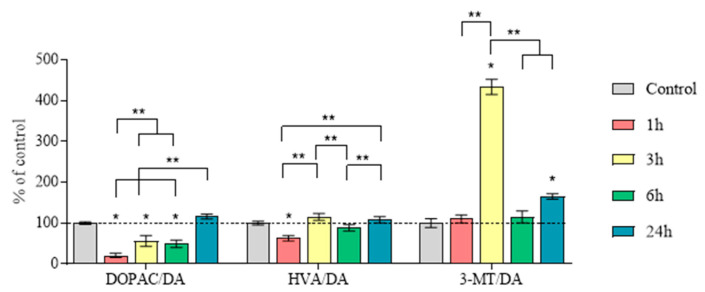
Dopamine (DA) turnover as a ratio of 3,4-dihydroxyphenylacetic acid/DA (DOPAC/DA), homovanillic acid/DA (HVA/DA), and 3-methoxytyramine/DA (3-MT/DA) (“*n*” per group = 8) in the striatum 1 h after saline injections (control) and 1, 3, 6, and 24 h after twice administration of 1-methyl-4-phenyl-1,2,3,6-tetrahydropyridine (MPTP) at a single dose of 6 mg/kg. Data are presented as mean ± SEM, with control taken as 100%. * *p* < 0.05 relative to the control group using the parametric Student’s test, ** *p* < 0.05 between selected groups using a one-way analysis of variance with Tukey’s test for post-analysis.

DA turnover, calculated as the DOPAC/DA ratio, was 20%, 56%, 50%, and 116% of the control levels at 1, 3, 6, and 24 h after MPTP administration, respectively (Figure 7, Table S2). Significant differences were found between all the experimental groups.

Based on the data obtained, DA turnover was also calculated as the HVA/DA ratio, which was 63% of the control 1 h after MPTP administration (Figure 7, Table S2). Three, six, and twenty-four h after MPTP administration, no changes in the HVA/DA ratio were noted, compared with the control. Significant differences were shown between the group collected 1 h after MPTP administration and the groups collected at 3 and 24 h. Significant differences were also shown between the group collected at 6 h and the groups collected at 3 and 24 h.

DA turnover, calculated as the 3-MT/DA ratio, was at the control level at 1 and 6 h after MPTP administration, while at 3 h, it was 424%, and at 24 h, it constituted 165% (Figure 7, Table S2). Significant differences were only shown between the 3 h group and all the other groups.

### 2.6. Characteristics of Tyrosine Hydroxylase in the Striatum

**Figure 8:**
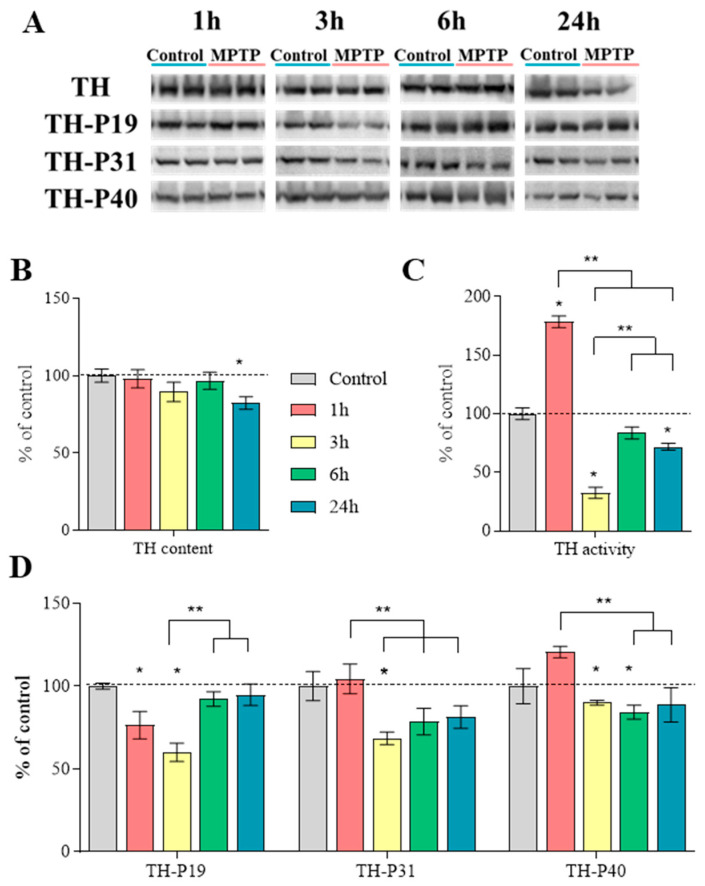
Characteristics of tyrosine hydroxylase (TH) in the striatum of mice 1 h after saline injections (control) and 1, 3, 6, and 24 h after twice administration of 1-methyl-4-phenyl-1,2,3,6-tetrahydropyridine (MPTP) at a dose of 6 mg/kg and in the control (0.9% NaCl): (**A**) representative Western blot of tyrosine hydroxylase (TH) and its phosphorylated forms at Ser19, Ser31, and Ser40; (**B**) TH content (“*n*” per group = 7); (**C**) TH activity, calculated as the difference in the l-3,4-dihydroxyphenylalanine (L-DOPA) content with and without inhibition of aromatic L-amino acid decarboxylase using 3-hydroxybenzylhydrazine (NSD-1015) (“*n*” per group = 8); (**D**) the content of phosphorylated forms of TH at Ser19, Ser31, and Ser40 in the striatum (“*n*” per group = 7). Data are presented as mean ± SEM, taken as 100% in the control. * *p* < 0.05 relative to the control group using the parametric Student’s test, ** *p* < 0.05 between selected groups using a one-way analysis of variance with Tukey’s test for post-analysis.

The TH content did not change at 1, 3, and 6 h after MPTP administration, while at 24 h, the TH content decreased by 18% compared with the control, taken as 100% (Figure 8A,B, Table S2).

TH activity, calculated as the difference in the L-DOPA content with and without inhibition of AADC, using NSD-1015, was 179% at 1 h after MPTP administration, 33% at 3 h, and 72% at 24 h compared with the control, taken as 100%. At 6 h, TH activity was at the control level (Figure 8C, Table S2). Significant differences were shown between the 1 h group and all the other groups.

The content of TH phosphorylated at Ser19 was reduced at 1 and 3 h after MPTP administration by 24% and 40%, respectively, and at 6 and 24 h, the content of this form of the protein was at the control level (Figure 8A,D, Table S2). Significant differences were shown between the group collected 3 h after MPTP administration and the groups collected at 6 and 24 h.

The content of TH phosphorylated at Ser31 did not change 1 h after MPTP administration, whereas at 3 h, it was significantly reduced by 32%, and at 6 and 24 h, there was a tendency for this parameter to decrease to 21% (*p* = 0.07) (Figure 8A,D, Table S2).

The content of TH phosphorylated at Ser40 did not change 1 and 24 h after MPTP administration compared with the control, and at 3 and 6 h, this parameter was reduced by 10% and 16%, respectively (Figure 8A,D, Table S2). Significant differences were shown between the group collected 1 h after MPTP administration and the groups collected at 3 and 6 h.

### 2.7. The Gene Expression of Proteins Involved in Dopamine Metabolism, Neurotransmission, Axonal Transport, Protein Degradation, Neuroprotection, and Neuron Death in the Substantia Nigra

In mice, 1 h after MPTP administration, there was an increase in the gene expression of proteins associated with the following: (i) DA synthesis, degradation, transport and reception (*Comt*); (ii) axonal transport and microtubules (*Mapt*, *Map2*); (iii) vesicle cycle for neurotransmission (*Syn1*, *Syt11*, *Dnm1l*, *Nsf*); (iv) protein degradation (*Psmd4*, *Psmb4*, *Usp47*); (v) neuroprotection (*Sod1*, *Gpx1*, *Txnrd1*, *Nfe2l2*, *Keap1*, *Ntrk2*); and (vi) inflammation and glial activation (*Cnr1*, *Clkk1*). There was a decrease in the gene expression of proteins associated with the following: (i) DA synthesis, degradation, transport, and reception (*Drd2*); (ii) axonal transport and microtubules (*Dynll1*); and (iii) neuroprotection (*Prdx1*). There was no change in the gene expression of proteins associated with the following: (i) DA synthesis, degradation, transport and reception (*Th*, *Ddc*, *Maoa*, *Maob*, *Slc6a3*, *Slc18a2*); (ii) axonal transport and microtubules (*Kif1a*, *Kif5a*, *Dctn1*, *Tubb3*, *Tuba1a*); (iii) vesicle cycle for neurotransmission (*Syn1*, *Syt1*, *Rab5a*, *Rabb7*); (iv) protein degradation *(Ube2n*, *Uba3*, *Psmc3*, *Ubb*, *Ctsb*); (v) neuroprotection (*Gsr*, *Sigmar1*, *Nr4a2*, *Calb1*); (vi) inflammation and glial activation (*Gfap*, *Akt1*); and (vii) apoptosis, necrosis, autophagy, and ER stress (*Parp1*, *Cib1*, *Aifm1*, *Bax*, *Mapk8*, *Lamp2*, *Atg16l1*, *Atg5*, *Trp35*) (Table 1).

Six hours after MPTP administration in mice, there was an increase in the gene expression of proteins associated with (i) DA synthesis, degradation, transport, and reception (*Maoa*); (ii) axonal transport and microtubules (*Map2*); (iii) vesicle cycle for neurotransmission (*Dnm1l*, *Nsf*); (iv) protein degradation (*Psmd4*, *Usp47*); (v) neuroprotection (*Txnrd1*, *Keap1*, *Ntrk2*); and (vi) inflammation and glial activation (*Cnr1*). There was a decrease in the gene expression of proteins associated with (i) DA synthesis, degradation, transport and reception (*Maob*, *Drd2*); (ii) axonal transport and microtubules (*Dynll1*); and (iii) protein degradation (*Ube2n*). There was no change in (i) DA synthesis, degradation, transport and reception (*Th*, *Ddc*, *Comt*, *Slc6a3*, *Slc18a2*); (ii) axonal transport and microtubules (*Kif1a*, *Kif5a*, *Dctn1*, *Mapt*, *Tubb3*, *Tuba1a*); (iii) vesicle cycle for neurotransmission (*Snca*, *Syn1*, *Syt1*, *Syt11*, *Rab5a*, *Rabb7*); (iv) protein degradation (*Uba3*, *Psmb4*, *Psmc3*, *Ubb*, *Ctsb*); (v) neuroprotection (*Sod1*, *Gpx1*, *Gsr*, *Prdx1*, *Nfe2l2*, *Sigmar1*, *Nr4a2*, *Calb1*); (vi) inflammation and glial activation (*Gfap*, *Akt1*, *Clk1*); and (vii) apoptosis, necrosis, autophagy, and ER stress (*Parp1*, *Cib1*, *Aifm1*, *Bax*, *Mapk8*, *Lamp2*, *Atg16l1*, *Atg5*, *Trp35*) (Table 1).

Twenty-four hours after MPTP administration in mice, there was an increase in the gene expression of proteins associated with (i) axonal transport and microtubules (*Map2*); (ii) vesicle cycle for neurotransmission (*Nsf*); (iii) protein degradation (*Usp47*); (iv) neuroprotection (*Txnrd1*, *Calb1*); and (v) inflammation and glial activation (*Cnr1*, *Clk1*). There was a decrease in the gene expression of proteins associated with (i) DA synthesis, degradation, transport, and reception (*Th*, *Slc6a3*, *Slc18a2*, *Drd2*); and (ii) the vesicle cycle for neurotransmission (*Snca*). There was no change in (i) DA synthesis, degradation, transport, and reception (*Ddc*, *Maoa*, *Maob*, *Comt*); (ii) axonal transport and microtubules (*Kif1a*, *Kif5a*, *Dynll1*, *Dctn1*, *Mapt*, *Tubb3*, *Tuba1a*); (iii) vesicle cycle for neurotransmission (*Syn1*, *Syt1*, *Syt11*, *Rab5a*, *Rabb7*, *Dnm1l*); (iv) protein degradation (*Ube2n*, *Uba3*, *Psmb4*, *Psmc3*, *Psmd4*, *Ubb*, *Ctsb*); (v) neuroprotection (*Sod1*, *Gpx1*, *Gsr*, *Prdx1*, *Nfe2l2*, *Keap1*, *Sigmar1*, *Ntrk2*, *Nr4a2*); (vi) inflammation and glial activation (*Gfap*, *Akt1*); and (vii) apoptosis, necrosis, autophagy, and ER stress (*Parp1*, *Aifm1*, *Cib1*, *Bax*, *Mapk8*, *Lamp2*, *Atg5*, *Trp35*) (Table 1).

## 3. Discussion

The first objective of this work was to develop a model of the earliest preclinical stage of PD to assess the initial molecular mechanisms of PD pathogenesis, including neurodegradation and neuroplasticity. There are two pathways for the development of PD: central, through the brain, and peripheral, through the intestine [21,22,23]. In our opinion, the central one is provoked by exogenous toxins (pesticides, heavy metals, etc.) in patients and MPTP in animals, entering the brain through the nasal part of the head along the cranial nerves [14,15]. In this case, pathological manifestations first appear in the olfactory bulbs, and then toxins cause the death of dopaminergic neurons without disrupting the metabolism of α-synuclein. However, when developing models of PD, scientists are guided by the fact that in patients with PD the transition from the preclinical stage to the clinical one is accompanied by the appearance of motor symptoms specific to PD, the death of nigral DAergic neurons, and a decrease in the DA concentration in the striatum by 70–80% [5,24,25,26,27]. There are three fundamentally different types of PD models: (i) genetic models based on the impaired metabolism of endogenous proteins, leading to their conversion into neurotoxins, such as α-synuclein; (ii) neurotoxic models based on the entry of exogenous neurotoxins—pesticides, heavy metals, etc.—into the brain; and (iii) combined models [10,14,28,29,30,31]. However, only some models using exogenous neurotoxins are able to meet the above requirements and reproduce the progressive stage-by-stage development of PD [17,31,32].

The most suitable and widely used exogenous neurotoxin for the above purpose is MPTP, which was originally obtained as a byproduct of the synthesis of desmethylprodine (1-methyl-4-phenyl-4-propionoxypiperidine), which causes parkinsonian symptoms in humans [2]. In animal models of PD via MPTP injections, it is possible to reproduce the progressive degradation of the nigrostriatal dopaminergic system, degradation of other monoaminergic systems of the brain, and peripheral nervous system, motor and non-motor symptoms, etc. The only significant drawback of MPTP models in rodents, but not in monkeys, is an absence of α-synuclein overproduction and aggregation [33,34,35]. This is confirmed by our many years of experience in using acute and subchronic mouse MPTP models of the advanced preclinical stage of PD [16,17]. In contrast to our previous studies, in this work, we collected materials for analysis shortly after the administration of MPTP—after 1, 3, 6, and 24 h, and not after 2 weeks, as was done previously [36,37,38]. In other words, we developed and used a model of the earliest preclinical stage of PD, suitable for identifying the initial molecular mechanisms of the pathogenesis of PD provoked by an exogenous specific neurotoxin of DAergic neurons. This model fully meets the requirements for simulating the preclinical stage of PD (see above): the animals did not show motor disorders, and there was no death of nigral DAergic neurons. In addition, the level of DA in the striatum decreased by 48%, without reaching the threshold value (70–80%) for the transition from the preclinical stage of PD to the clinical stage [13]. However, experimental data should be translated to patients with caution, since the acute and chronic modeling PD using single or repeated administration of MPTP, respectively, does not accurately reproduce the monotonous change in the concentration of exogenous and endogenous toxins in patients as PD progresses.

In addition to a decrease in dopamine concentrations in the striatum of MPTP-treated mice, we found a 26% decrease in the number of DAergic (TH-, AADC-, and DAT-immunopositive) fibers (varicosities) [39], but only 24 h after injection of the toxin. The degradation of DAergic fibers without changing the number of corresponding neurons in the SN confirms the concept of retrograde neuronal degeneration due to the higher sensitivity of axonal terminals compared to neuronal cell bodies to MPP^+^ [9,40,41]. In fact, this is explained by the greater number of DAT molecules that capture MPP^+^ in the axon membrane than in the membrane of the neuronal cell body [42].

In addition to DA, we assessed the level of MPP^+^ in the striatum and SN, which is another important characteristic of our PD model. The change in MPP^+^ concentration in the interval from 1 to 24 h after MPTP administration was similar in the striatum and in the SN. 1 h after MPTP administration, high levels of MPP^+^ were observed in both the striatum and the SN. After 3 and 6 h, this index decreased by three times, and after 24 h there was a 20-fold decrease in the level of MPP^+^ in the striatum and a 40-fold decrease in the SN. This timing characteristic of MPP^+^ incorporation into the nigrostriatal system is in agreement with previous studies [19,20,43,44]. The maximum incorporation of MPP^+^ into the nigrostriatal system, observed 1 h after MPTP administration appears to be due to the initial uptake of MPTP by glial cells, where it is converted by monoamine oxidase B (MAO B) to MPP^+^ [45]. A further two-step decrease in the level of MPP^+^ in the striatum and SN (at 3 and 6 h) is apparently associated with the capture of MPP^+^ secreted by glial cells into DAergic neurons (cell bodies and processes) via the DAT and vesicular monoamine transporter 2 (VMAT2), followed by its removal from nigral DAergic neurons and their axons in the striatum by the end of the studied period (24 h) [19,20,43,44].

The key objective of this work was to study the initial molecular mechanisms of degradation in the nigrostriatal DAergic system under the influence of MPTP, since they can be considered as targets for developing new medicines and improving the current treatment of PD. The main focus of our research was the striatum, a key brain structure involved in DA regulation of motor function [46]. It should be noted that the DA contained in the striatum is synthesized not only in the DAergic axons of nigral neurons, but also in the striatal neurons expressing enzymes for DA synthesis [36,47]. According to previous studies, DAergic deafferentation of the striatum in parkinsonian animals results in an increase in the number of striatal neurons expressing DA-synthesizing enzymes, including TH, as well as the proportion of DA synthesis by these neurons in the total DA synthesis in the striatum [36,48,49,50,51,52].

It is tempting to differentially assess the contribution to the DA content in the striatum, and hence, the regulation of motor behavior, made by the DA-synthesizing neurons of the striatum and the DAergic axons of nigral neurons. However, this problem cannot currently be solved for technical reasons. Indeed, this requires the development of a method for sorting striatal neurons expressing DA-synthesizing enzymes and synaptosomes of nigral DAergic neurons. Therefore, to assess early changes in DA metabolism in the striatum of mice in our model of the earliest preclinical stage of PD, we used an integral index of the chemical machinery involved in DA synthesis, with an emphasis on changes in both the content (synthesis) and activity of TH, the rate-limiting enzyme of DA synthesis [53].

To assess the functional state of the chemical machinery for DA synthesis, we measured the content of TH, its activity, and the content of TH phosphorylated at three sites on serines: Ser19 (TH-P19), Ser31 (TH-P31), and Ser40 (TH-P40). Phosphorylation at Ser19 is an indicator of subsequent proteasomal degradation of TH [54,55], whereas phosphorylation at Ser40 and Ser31 leads to increased TH activity [56,57].

It was shown that the total TH content in the striatum does not change for 6 h after administration of MPTP, which is apparently due to a simultaneous decrease in the content of TH-P19, responsible for the compensatory stabilization of TH. Only after 24 h, the TH content decreases, accompanied by an increase in the content of TH-P19 and a decrease in the TH gene expression in DAergic neurons in the SN (Table 1).

While the TH content in the striatum remains at the control level for at least 6 h after administration of MPTP, TH activity in this brain region changes significantly during the entire studied period compared to the control. Indeed, 1 h after MPTP administration, TH activity increased compared to the control, which apparently is a compensatory reaction associated with a tendency to increase the TH-P40 content (*p* = 0.07). Maintaining TH activity at the control level 6 h after administration of MPTP can also be considered as a compensatory reaction that develops due to an increase in the level of TH-P31. On the contrary, we consider the decrease in TH activity observed 3 and 24 h after MPTP administration as a manifestation of the degradation of the DA-producing striatal neurons and the DAergic axons of nigral neurons. The largest decrease in total TH activity observed 3 h after MPTP administration appears to be associated with a decrease in the concentrations of TH-P31 and TH-P40 (see Results). Previously, we observed an even greater decrease in TH activity, as well as in the content of TH-P31 and TH-P40 in the striatum of mice in a model of the clinical stage of PD, without their subsequent restoring [58]. Thus, regulation of the synthesis and activity of phosphorylated TH can be considered as a potential target for improving the treatment of PD.

As to the regulation of motor function, the most important manifestation of neurodegradation and neuroplasticity of the striatum is the concentration of DA, which is the resultant of three associated processes: synthesis, release, and degradation of DA. From the observed changes in the concentration of the main products of enzymatic degradation of DA—DOPAC and HVA—in the striatum, it follows that DA degradation decreases throughout the entire studied period after MPTP administration. We consider this fact as a compensatory reaction aimed at minimizing the loss of DA in the striatum following its DAergic deafferentation.

However, even with a compensatory decrease in DA degradation in the striatum, a gradual decrease in DA concentration was observed over a 3–24 h period after MPTP administration, which could hypothetically be associated with either a gradual decrease in the number of varicose swellings of nerve fibers, DA synthesis, or/and a gradual increase in its release. A number of data appear to support the idea of decreased DA synthesis in the striatum following MPTP exposure: (i) decreased TH gene expression in nigral neurons, (ii) decreased TH content 24 h after MPTP exposure, and (iii) decreased TH activity due to a decrease in the level of its phosphorylated forms in the striatum from 3 to 24 h after MPTP administration. This issue should be resolved in further work using the method we have developed for differential assessment of the synthesis and release of DA during the incubation of thick sections of nervous tissue containing DA-secreting neurons [37,59].

With regard to the release of DA, we have obtained indirect evidence of an increase in its release from striatal DAergic fibers after partial DAergic deafferentation of this brain region. Indeed, 3 h after MPTP administration, we observed a significant increase in the concentration of 3-MT in the striatum, a product of DA conversion by catechol-O-methyltransferase (COMT), localized in the extracellular space and in glial cells [60,61]. These data are consistent with findings from the literature indicating a decrease in stimulated DA and an increase in non-stimulated DA release from striatal slices when incubated in the presence of MPP^+^ [62]. The hypothetical increase in DA release from DAergic fibers 3 h after MPTP administration is probably aimed at compensatory prevention of the accumulation of cytosolic DA, which becomes cytotoxic in high concentrations [63]. With regard to the release of DA, we have obtained indirect evidence of an increase in its release from striatal DAergic fibers after partial DAergic deafferentation of this brain region. Indeed, 1 h after MPTP administration, we observed a significant increase in *Comt* expression in the SN and at 3 h after MPTP injection, we observed an increase in the concentration of 3-MT in the striatum, a product of DA conversion by COMT, localized in the extracellular space and in glial cells [60,61]. These data are consistent with the literature, indicating a decrease in stimulated DA and an increase in non-stimulated DA release from striatal slices when incubated in the presence of MPP^+^ [62,64,65]. The increase in DA release from DAergic fibers 3 h after MPTP administration is probably aimed at compensatory prevention of the accumulation of cytosolic DA, which becomes cytotoxic in high concentrations [63].

It is believed that the integral index of the efficiency of DA neurotransmission is DA turnover, which is calculated as the ratio of the concentration of one of the DA degradation products to the concentration of DA itself [66,67]. Given the different localization of DA degradation enzymes—COMT in the extracellular space and in glial cells [68,69] and monoamine oxidase A (MAO A) in neurons [70]—DOPAC/DA is an indicator of DA turnover inside neurons, and 3-MT/DA is an indicator of DA turnover in the extraneuronal space. Since HVA is the end product of DA degradation, HVA/DA is an indicator of the total DA turnover. When calculating DA turnover using DOPAC or HVA as degradation products, it is evident that in both cases, DA turnover was reduced an 1 h after MPTP administration. However, in the first case (DOPAC/DA), DA turnover compensatorily increases to the control level 24 h after the administration of MPTP, and in the second case (HVA/DA), after 3 h. The increase in DOPAC/DA turnover is associated with an increase in DOPAC concentration due an increase *Maoa* expression 6 h after MPTP administration. These data are partly consistent with previous studies that showed that DA turnover in the striatum was reduced when using other regimes of MPTP administration for modeling the preclinical stage of PD [58,71]. Given that DOPAC is the major intraneuronal product of DA degradation, DA turnover, expressed as DOPAC/DA, is the main index of the efficiency of DA neurotransmission [68,69].

Although it is impossible to differentially assess the contribution of striatal neurons and DAergic axons of nigral neurons to the total content (synthesis) of DA in the striatum (see above), we attempted to estimate, at least indirectly, the contribution of the latter to the synthesis of DA in the striatum. TH activity and DA content were chosen as the main indexes of the functional state of nigral DAergic neurons. However, we did not see a correlation in changes in the SN and in the striatum for any of these indexes. Indeed, TH activity in the SN first decreases (1, 3, 6 h), and then (24 h) compensatorily returns to the control level, whereas in the striatum, TH activity increases sharply compared to the control 1 h after MPTP administration, followed by its drop after 3 h. Although TH activity in the striatum increases to the control level 6 h after MPTP administration, it remains below the control level at 24 h.

When assessing the content of DA and TH activity in the SN, no correlation was found with changes in the DA concentration in the striatum. Indeed, the DA content in the SN 1, 3, and 6 h after MPTP administration significantly decreased compared to the control, and only after 24 h it compensatorily increased to the control level. In turn, the DA concentration in the striatum gradually decreased, starting 3 h after MPTP administration. The lack of visible synchronization in DA metabolism in the striatum and in nigral DAergic neurons is apparently due to differences in the functional role of DA and the regulation of its secretion by intercellular signals in these regions. Indeed, the primary role of the DA secreted by the DAergic neurons in the SN is to ensure the regulation of nigral target neurons by this neurotransmitter, including autoregulation [72]. 

Although our attempts to assess the contribution of nigral DAergic neurons (neuron cell bodies) to the maintenance of DA homeostasis in the striatum after its DAergic denervation were not successful, the molecular mechanisms of neurodegradation and neuroplasticity in the SN that we identified can serve as targets for the development of new medicines and the improvement of the current treatment of PD. In further studies, the list of these targets can be significantly expanded by taking into account our data on changes in gene expression in nigral neurons 1, 6, and 24 h after MPTP administration (see Table 1). Particular attention should be paid to a decrease in gene expression of such functionally significant proteins as TH, DAT, and VMAT2. Similar changes in DAT and TH gene expression were previously shown in another model of preclinical PD [73]. As our previous studies have shown, changes in the expression of some genes of proteins of the DAergic phenotype (TH, DAT) found in this work are leveled out over time (2 weeks after MPTP administration of) [37,59,74].

In contrast to the expression of the TH, DAT, and VMAT2 genes, the expression of the *Drd2* gene, encoding the autoreceptor DA type 2 [75], was reduced 1 h after MPTP administration, and it subsequently remained at this level. If we assume that the expression of the *Drd2* gene correlates with protein content, as in the case of TH, then the changes we discovered reflect a compensatory increase in the activity of DAergic neurons after the toxic action of MPTP. We also observed a reduced expression of *Drd2* in a previous study in mice using a subchronic model during the preclinical stage, but not the clinical stage, of PD [17]. This suggests that a decreased expression of D2 receptors is characteristic of the preclinical stage of PD.

We also obtained data suggesting a compensatory increase in DA neurotransmission in a model of the early preclinical stage of PD. Thus, 1 h after the administration of MPTP, the expression of some synaptic protein genes increased: synapsin 1 (*Syn1*) and synaptotagmin 11 (*Syt11*). In addition, the expression of the N-ethylmaleimide sensitive fusion protein (*Nsf*) gene increased throughout the studied period. The participation of these proteins in neurotransmission is that synapsin 1 is involved in the transport of synaptic vesicles and the regulation of neurotransmitter release [76], synaptotagmin 11 is involved in the regulation of endocytosis [77], and the N-ethylmaleimide sensitive fusion protein is involved in the disassembly of the SNARE complex after vesicular fusion with the plasma membrane of the neuron [78]. The assumption of increased neurotransmission is also confirmed by the fact that the concentration of 3-MT is increased in the striatum 3 h after MPTP administration.

According to our data, 24 h after MPTP administration, the expression of the *Snca* gene, which encodes another synaptic protein—α-synuclein—decreased. It is known that mutations in the α-synuclein gene lead to the development of PD, and its accumulation in Lewy bodies is a key element in the pathogenesis of the disease [79,80,81,82,83]. Considering that changes in *Snca* gene expression occur later than in the genes of other synaptic proteins we studied, the function of α-synuclein is not limited to the regulation of the pool of synaptic vesicles and their transport [84]. Indeed, α-synuclein is involved in maintaining calcium homeostasis in mitochondria [85]. It is important to note that calbindin is also involved in maintaining calcium homeostasis in cells [86], and the gene expression of this protein, as α-synuclein, increases only 24 h after MPTP administration.

In contrast to monkeys, rodents treated with MPTP do not generate Lewy bodies, which consist mainly of α-synuclein aggregates [33,34,35]. In this context, the decrease in *Snca* gene expression that we found in mice exposed to MPTP may be a compensatory process aimed at reducing α-synuclein synthesis and aggregation.

In addition to the genes mentioned above, we also assessed the expression of genes of proteins involved in axonal transport—kinesin (anterograde transport), dynein (retrograde transport), microtubule proteins (α- and β-tubulin), and microtubule stabilizer proteins (tau protein, MAP2) [87,88,89]. According to our data, after the administration of MPTP, the expression of tubulin protein genes did not change during the studied period, but the expression of the tau (*Mapt*) and MAP2 (*Map2*) protein genes increased, and the expression of the dynein protein gene (*Dynll1*) decreased. An increase in the content of stable forms of tubulin (acetylated and detyrosinated) after administration of MPTP/MPP^+^ in vitro and in vivo was previously shown [71,90], which is consistent with the increased content of tau protein and MAP2 mRNA that we found. But despite this, the rate of anterograde transport decreases, while retrograde transport increases after the addition of MPTP [71,91,92]. The slowing of anterograde transport has also been shown in cases of tau protein overexpression [93,94]. An interpretation of our results on the gene expression mentioned above is difficult without further testing of axonal transport in a model of the early preclinical stage of PD.

We also paid considerable attention to the expression of protein genes involved in protein degradation through both the ubiquitin–proteasome system and the autophagy–lysosomal pathway, which requires modification of the target with ubiquitin [95]. According to the data obtained, the expression of the ubiquitin gene did not change in the first 24 h after MPTP administration. However, multidirectional changes in gene expression were observed: an increase in the expression of the genes for the proteasome subunit *Psmb4* (1 h after administration of MPTP) and *Psmd4* (1 and 6 h after administration of MPTP) and a decrease in the mRNA of the ubiquitin-conjugating enzyme (*Ube2n*, 6 h after administration of MPTP). This study should be further developed, since the ubiquitin-conjugating enzyme is involved not only in the ubiquitination of proteins to ensure their utilization but also in parkin-mediated mitophagy [96].

It should be noted that, during the entire period we studied after exposure to MPTP, the expression of the *Usp47* gene, encoding a protein that carries out the deubiquitination of proteins, increased [97]. In PD, it has been shown that the expression of the *Usp47* gene, as well as that of the genes of many proteins in the ubiquitin–proteasome system, is reduced in the SN [98,99]. However, these data were obtained in postmortem patients many years after impaired motor function, with an almost complete loss of DAergic neurons in the nigrostriatal system and depletion of compensatory reserves of the brain. These data suggest an increase in the lifetime of proteins as one of the compensatory mechanisms of the preclinical stage.

The toxic effect of MPP^+^ on DAergic neurons is promoted, on the one hand, by its penetration into mitochondria with subsequent inhibition of complex I in the respiratory chain, ATP depletion, and the formation of reactive oxygen species (ROS) [100]. On the other hand, it is promoted by an increase in the level of cytosolic DA as a result of the accumulation of MPP^+^ in synaptic vesicles. Cytosolic DA is rapidly oxidized to quinones, which also leads to an increase in ROS levels. Indeed, in this work, we found an increase in the expression of the *Dnm1l* gene, which encodes the protein dynamin-related protein 1. It is involved in mitochondrial fission and mitophagy [101]. It has been shown that a decrease in the expression of this protein leads to a decrease in the toxic effect of MPTP and the restoration of DA release in the striatum [102]. The increase in *Dnm1l* expression shown in this work is probably associated with an increased autophagy of damaged mitochondria under the toxic effect of MPP^+^.

It is known that to protect cells from ROS, the antioxidant system—a complex of enzymes that can restore the redox status of the cells—is used. One of the regulators of the antioxidant system is the transcription factor Nrf2 (*Nfe2l2*), which is normally associated with Keap1 and is localized in the cytoplasm [103]. When the level of ROS increases, the above complex is destroyed, and Nrf2 is transported to the nucleus, where it triggers the transcription of enzymes in the antioxidant system [104]. Activation of the antioxidant system is a key mechanism for protecting neurons from the toxic effect of MPP^+^. According to our data, 1 h after MPTP administration, the expression of the *Nfe2l2* gene and that of the *Sod1*, *Gpx1*, and *Txnrd1* genes, encoding superoxide dismutase 1, glutathione peroxidase 1, and thioredoxin reductase 1, respectively, increase. After 6 h, only *Txnrd1* and *Keap1* showed increased expression, while the expression of the remaining genes decreased to control values. This correlates with the enzyme content in the midbrain of mice after MPTP administration [105].

As we expected, since all SN DAergic neurons survived after MPTP administration, the gene expression of the proteins involved in cell death did not change, at least during the studied period (Table S3).

Thus, the data obtained in this work indicate large compensatory reserves of the brain at the earliest preclinical stage of PD, which opens up broad prospects for studying the molecular mechanisms of neuroplasticity and developing preventive neuroprotective treatments.

## 4. Materials and Methods

### 4.1. Animals and Experimental Procedures

In these experiments, we used male mice of the C57BL/6 line (*n* = 204), aged 8–12 weeks and with a body weight of 20–24 g, obtained from the Stolbovaya nursery (SCBMT RAMS, Stolbovaya, Moscow reg., Russia). The animals were kept at a temperature of 22 ± 1 °C, with a 12 h day/night cycle and free access to food and water. The experimental procedures were carried out in accordance with the National Institutes of Health Guide for the Care and Use of Laboratory Animals (8th edition, 2011) and were approved by the Animal Care and Use Committee at the Koltzov Institute of Developmental Biology, Russian Academy of Sciences (protocol No. 50 from 5 August 2021).

In the first series of experiments, experimental group animals (*n* = 60), used to model the early preclinical stage of PD, were injected twice with MPTP (Sigma-Aldrich, Saint Louis, MO, USA) at a dose of 6 mg/kg subcutaneously at the withers, with a 2 h interval between injections. The regimen was chosen based on a previously developed model of the preclinical stage of PD (two MPTP administrations at a dose of 12 mg/kg) [16]. Mice in the control group (*n* = 60) were subcutaneously injected with a 0.9% NaCl solution according to a similar scheme.

In the second series of experiments, animals were administered subcutaneously (at the withers) with MPTP (*n* = 42) or 0.9% NaCl solution (*n* = 42) according to the scheme described above. Each group was then randomly divided into 2 equal subgroups. Animals in the first subgroup were administered an AADC inhibitor, 3-hydroxybenzylhydrazine (NSD-1015, Sigma-Aldrich, Saint Louis, MO, USA), intraperitoneally at a dose of 100 mg/kg body weight [106], half an h before decapitation, whereas in the second subgroup, a 0.9% NaCl solution was administered. Animals in the first subgroup were administered an AADC inhibitor, 3-hydroxybenzylhydrazine (NSD-1015, Sigma-Aldrich, Saint Louis, MO, USA), intraperitoneally at a dose of 100 mg/kg body weight [106], half an h before decapitation, whereas in the second subgroup, a 0.9% NaCl solution was administered.

### 4.2. Sample Preparation

**Figure 9:**
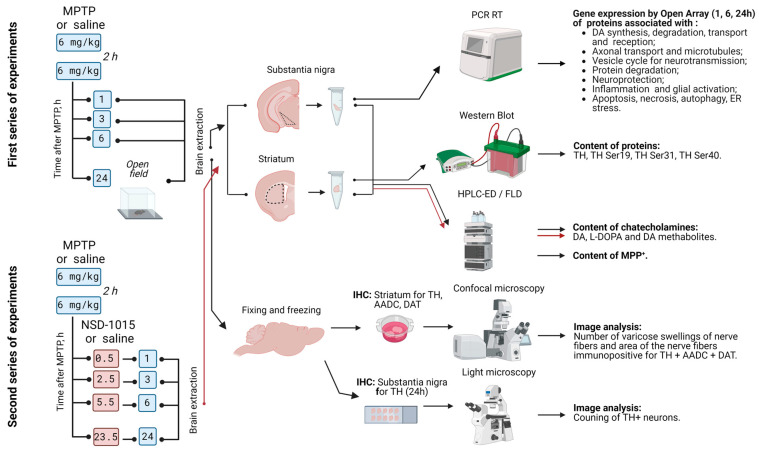
Scheme of experiments with subcutaneous administration of 1-methyl-4-phenyl-1,2,3,6-tetrahydropyridine (MPTP) to mice twice at a dose of 6 mg/kg with an interval of 2 h, followed by obtaining the striatum and substantia nigra 1, 3, 6, and 24 h after the last injection and the subsequent processing and analysis of samples. AADC—aromatic L-amino acid decarboxylase; DA—dopamine; DA metabolites: 3-MT—3-methoxytyramine, DOPAC—3,4-dihydroxyphenylacetic acid, HVA—homovanillic acid; DAT—dopamine transporter; ER—endoplasmic reticulum; HPLC-ED/FLD—high-performance liquid chromatography with electrochemical and fluorescence detections; IHC—immunohistochemistry; L-DOPA—L-3,4-dihydroxyphenylalanine; MPP^+^—1-methyl-4-phenylpyridinium; MPTP—1-methyl-4-phenyl-1,2,3,6-tetrahydropyridine; NSD-1015—3-hydroxybenzylhydrazine; TH—tyrosine hydroxylase.

In the first and second series of experiments, the material was collected 1, 3, 6, and 24 h after the last injection of MPTP or 0.9% NaCl (Figure 9). Mice in the experimental and control groups were decapitated under anesthesia with 2.4% isoflurane (Baxter, Deerfield, IL, USA). The brain was removed from the skull and cut along the midsagittal plane at +4 °C. The striatum from one half of the brain was dissected under visual control using a Leica M60 dissecting microscope (Leica, Wetzlar, Germany), as described previously [16,59]. The resulting samples were placed into a test tube, weighed on a Mettler AT201 analytical balance (Mettler Toledo, Greifensee, Schweiz), frozen in liquid nitrogen, and stored at −70 °C until further analysis of the material.

The material (striatum and SN) obtained in the first series of experiments was used to determine the content of (a) DA and its metabolites, including DOPAC, 3-MT, and HVA, using high-performance liquid chromatography with electrochemical detection (HPLC with ED) (“*n*” per group = 8); (b) MPP^+^, using HPLC with fluorescence detection (“*n*” per group = 8); (c) TH and its phosphorylated forms according to Ser19, Ser30, and Ser40 using Western blot (“*n*” per group = 7); and (d) gene expression, using RT-PCR (“*n*” per group = 7) and Open Array technology (“*n*” per group = 6). The material (striatum and SN) obtained in the second series of experiments was used to determine the content of L-DOPA using HPLC-ED (“*n*” per group = 7).

The second half of the brain, obtained from the animals 24 h after MPTP injections in the first series of experiments, was fixed by immersion in 4% paraformaldehyde (Sigma-Aldrich, Saint Louis, MO, USA), prepared in a 0.2 M phosphate buffer (pH = 7.2–7.4) for 12 h at 4 °C. The samples were then washed in 0.02 M phosphate-buffered saline (PBS) (0.9% NaCl in 0.02 M phosphate buffer) (pH = 7.2–7.4), incubated in 20% sucrose in 0.02 M PBS for 48 h, and frozen in hexane cooled to −40 °C. The samples were stored at −70 °C until immunostaining of TH in SN sections (24 h after MPTP injections) and AADC, DAT, and TH in striatum sections (1, 3, 6 and 24 h after MPTP injections) (“*n*” per group = 3).

### 4.3. Motor Behavior of Mice

In the first series of experiments, the animals that were decapitated 24 h after the last injection of MPTP or 0.9% NaCl were preliminarily distributed into groups (control and experiment), based on an assessment of their motor behavior. Motor behavior—the distance traveled and the number of rearings—was recorded for 6 min in the “open field” test using an automated PhenoMaster setup (TSE Systems, Berlin, Germany). Based on the results obtained, the mice were divided into groups, so that the average distance traveled was the same, and the number of rearings did not differ between the animals of the two groups. After 23.5 h following the last injection of MPTP or 0.9% NaCl, the animals were reassessed for their motor behavior in the open field test.

### 4.4. Immunohistochemistry

On a cryostat (Leica CM1950, Leica, Wetzlar, Germany), in accordance with the mouse brain atlas [107], frontal SN sections with a thickness of 20 μm were prepared (from −2.70 mm to −3.88 mm relative to bregma) and mounted on glass slides. For immunohistochemical detection of antigens, every 6th section of the SN was taken. On a cryostat (Leica CM1950, Leica, Wetzlar, Germany), in accordance with the mouse brain atlas [107], frontal SN sections with a thickness of 20 μm were prepared (from −2.70 mm to −3.88 mm relative to bregma) and mounted on glass slides. For immunohistochemical detection of antigens, every 6th section of the SN was taken.

The SN sections were sequentially incubated with (i) 3% bovine serum albumin (BSA) (Sigma-Aldrich, Saint Louis, MO, USA) and 0.3% Triton X-100 (Triton-X100, Sigma-Aldrich, Saint Louis, MO, USA) in PBS, for 30 min; (ii) sheep anti-TH antibodies (1:700, ab1542, Sigma-Aldrich, Saint Louis, MO, USA), 1% BSA, and 0.1% Triton X-100 in PBS, for 20 h; (iii) biotinylated goat anti-sheep antibodies (1:200) (Vector Laboratories, Newark, CA, USA) in PBS, for 2 h; and (iv) avidin-biotin complex coupled with horseradish peroxidase (Vector Laboratories, Newark, CA, USA) in PBS, for 1 h. After each incubation, except for the first, the sections were washed in PBS for 30 min. All incubations were done at 20 °C. The sections were placed into a Mowiol 4-88 hydrophilic medium (Sigma-Aldrich, Saint Louis, MO, USA).

The striatum sections were sequentially incubated in PBS containing (i) 1% sodium dodecyl sulfate (Sigma-Aldrich, St. Louis, MO, USA) for 5 min; (ii) 3% bovine serum albumin (BSA, Sigma-Aldrich, St. Louis, MO, USA) and 0.3% Triton X-100 (Sigma-Aldrich, St. Louis, MO, USA) for 1 h; (iii) sheep polyclonal anti-TH antibodies (1:1000, ab1542, Millipore, Burlington, MA, USA) and rabbit polyclonal anti-AADC antibodies (1:300, ab3905, Abcam, Cambridge, UK), 1% BSA, and 0.1% Triton X-100 for 20 h; (iv) donkey anti-rabbit gamma globulin Alexa Fluor 555 antibodies (1:1000, A32794, Invitrogen, Thermo Fisher Scientific, Waltham, MA, USA) and donkey anti-sheep gamma globulin Alexa Fluor 633 antibodies (1:1000, A21100, Invitrogen, Thermo Fisher Scientific, Waltham, MA, USA) for 2 h; (v) rat monoclonal anti-DAT antibodies (1:500, MAB369, Millipore, Burlington, MA, USA) for 20 h; and (vi) donkey anti-rat gamma globulin CF™ 633 antibodies (1:500, SAB4600133, Sigma-Aldrich, St. Louis, MO, USA), in PBS, for 2 h. After each incubation, except for the second, the sections were washed three times in PBS for a total of 45 min. All incubations were done at 20 °C. The sections were placed into a medium containing DAPI (Abcam).

### 4.5. Microscopy

After the immunohistochemical detection of TH, the SN sections were studied under an Olympus BX51 light microscope (Olympus Corporation, Tokyo, Japan) equipped with an Olympus DP70 digital camera (Olympus Corporation, Tokyo, Japan), with an objective magnification of 10× and 20×. In ImageJ 1.53t (Fiji) software (https://imagej.net/software/fiji/downloads, accessed on 15 December 2023, USA), the compact part of the SN was outlined in the image of each section. Within the outlined compact part of the SN, only the cell bodies of neurons with a visible nucleus were counted. The total number of neurons in the entire SN was determined using the approximation method [108]. 

Sections of the striatum after immunostaining for TH, DAA, and DAT were studied under a Zeiss LSM 880 confocal microscope (Zeiss AG, Jena, Germany) (Center for Advanced Study, IBR RAS). From each section, 4 z-stacks were made in 4 regions of the striatum (the dorsomedial, dorsolateral, ventromedial, and ventrolateral parts) [109]. We alternately scanned in three channels to avoid the “exposure” of the red channel. The optical cutting step was 0.2 µm. Sections of the striatum after immunohistochemical detection of TH, AADC, and DAT were studied under a Zeiss LSM 880 confocal microscope (Zeiss AG, Jena, Germany) (Center for Advanced Study, IBR RAS). From each section, 4 z-stacks were made in 4 regions of the striatum (the dorsomedial, dorsolateral, ventromedial and ventrolateral parts) [109]. We alternately scanned in three channels to avoid the “exposure” of the red channel. The optical cutting step was 0.2 µm.

Using the Fiji program, varicosities containing only TH or AADC, and those containing TH, AADC, and DAT, as well as the total area of their fibers were counted in the image [36]. The algorithm contained the following steps: converting images to 8-bit, normalizing images inside the slice to the overall brightness of the images, determining the fluorescence signal threshold, and applying the filter median = 1. Using the AND function, images were obtained that contained fibers and varicosities swelling that are immune-immunopositive for TH, AADC, and DAT. Next, a watershed and conversion to a mask were applied to the created images, followed by analyzing particles with a choice of sizes (area) for varicose swellings ranging from 0.3 to 7 μm^2^ and the area of nerve fibers containing TH, AADC, and DAT in the striatum per 100 μm^2^.

### 4.6. High-Performance Liquid Chromatography with Electrochemical and Fluorescent Detection

In the samples of striatum and SN, the content of DA, L-DOPA, HVA, and 3-MT (only striatum) was determined using the HPLC-ED method. Additionally, L-DOPA was determined, using the same method, in the striatum and SN before and after inhibition of AADC using NSD-1015. The samples were homogenized using a Labsonic M ultrasonic homogenizer (Sartorius, Göttingen, France) in 0.1 N HClO_4_ (Sigma-Aldrich, Saint Louis, MO, USA) containing 250 pmol/mL of the internal standard: 3,4-dihydroxybenzylamine hydrobromide (Sigma-Aldrich, Saint Louis, MO, USA). The homogenate was centrifuged for 20 min at 2000× *g*.

Separation of L-DOPA, as well as of the DA and its metabolites, was carried out on a ReproSil-Pur, ODS-3, 4 × 100 mm reverse-phase column with a pore diameter of 3 μm (Dr. Majsch, Ammerbuch, Germany) at 30 °C and a mobile phase speed of 1 mL/min on a liquid chromatograph LC-20ADsp (Shimadzu Corp., Kyoto, Japan). The mobile phase consisted of a 0.1 M citrate-phosphate buffer, 0.3 mM sodium octanesulfonate, 0.1 mM EDTA, and 8% acetonitrile (all reagents from Sigma, USA), pH 2.5. The Decade II electrochemical detector (Antec Scientific, Alphen aan den Rijn, The Netherlands) was equipped with a glassy carbon working electrode (+0.85 V) and an Ag/AgCl reference electrode. The peaks of interest and the internal standard were identified by their appearance time in the standard solution. The content of DA and its metabolites were calculated by the internal standard method using a calibration curve in LabSolutions v 5.87 software (Shimadzu Corp., Kyoto, Japan). The content of DA, its metabolites, and L-DOPA in the striatum after AADC inhibition were normalized to tissue weight since these substances are evenly distributed throughout the excised tissues. The content of DA and its metabolites in the substantia nigra were not normalized to tissue weight because they are usually excised with varying volumes of surrounding tissue, which does not contain dopaminergic neurons.

In the same tissue samples (substantia nigra and striatum), the MPP^+^ concentration was also determined via HPLC with fluorescence detection. The excitation/emission wavelength was 310/360 nm.

Based on the data obtained, DA turnover was determined as the ratio of the content/concentration of DA metabolites (DOPAC, 3-MT, or HVA) to DA in the SN and striatum, respectively.

### 4.7. Assessment of Enzymatic Activity of Tyrosine Hydroxylase

TH activity in the striatum and the SN was determined as the difference in the L-DOPA content in samples, with and without administration of the AADC inhibitor NSD-1015.

### 4.8. RT PCR

Isolation of total RNA from mouse SN samples 24 h after MPTP administration was carried out in 1 mL of TRI reagent (Sigma-Aldrich, Saint Louis, MO, USA) according to the manufacturer’s instructions. To improve RNA precipitation, 1 μg of glycogen (Thermo Fisher Scientific, Waltham, MA, USA) was added to each sample. The concentration of total RNA in the samples was determined using a NanoDrop 8000 spectrophotometer (Thermo Fisher Scientific, Waltham, MA, USA). Total RNA was treated with RNase-free DNase I (Thermo Fisher Scientific, Waltham, MA, USA) to remove residual genomic DNA. For reverse transcription, 0.5 μg of RNA from the SN samples was used. Reverse transcription was performed using the RevertAid H Minus First Strand cDNA synthesis kit and random hexamer primers according to the manufacturer’s recommendations (Thermo Fisher Scientific, Waltham, MA, USA): the reaction was carried out for 60 min at 42 °C and stopped by heating for 10 min at 70 °C, followed by cooling the samples on ice.

RT-PCR was performed using the Open Array technology on TaqMan Open Array RT-PCR Custom Format 112 chips (Lot 37B6745, REF 4470756, Applied Biosystems, San Francisco, MA, USA), which made it possible to simultaneously determine the expression of 102 genes of interest in each sample (Table 2 and Table S1). For Open Array PCR, 250 ng cDNA was used for 56-well segments.

The results were processed using QuantStudio 12K Flex (Applied Biosystems, MA, USA) and Excel version 2312 (Microsoft Office 365, Redmond, WA, USA) software. Gene expression was assessed via the 2^−ΔΔCt^ method, using *Cyc1* as the housekeeping gene [110]. To calculate ΔΔCt, the following formulas, Equations (1) and (2), were used:

ΔΔCt = (ΔCt (sample) − ΔCt (control average))(1)

where

ΔCt = (Ct (gene) − Ct (*Cyc1*)) (2)

The results were calculated as the geometric mean of the group [111] and were presented as the fold change compared with the control, where the ΔΔCt of the control group was taken as 1. 

The analysis included genes for which the amplification curve crossed the detection threshold earlier than cycle 28 [112,113]. 

The gene names were taken from the GenBank database of the National Library of Medicine (https://www.ncbi.nlm.nih.gov/genbank, accessed on 15 November 2022).

### 4.9. Western Blot

The striatum samples were homogenized in RIPA buffer with a 2× proteinase inhibitor cocktail (Thermo Fischer Scientific, Waltham, MA, USA) and a 1× phosphatase inhibitor cocktail (Cell Signalings, Danvers, MA, USA) in 300 µL and were centrifuged for 20 min at 20,000× *g* and 4 °C. The protein concentration was determined using Bicinchoninic Acid Solution (BCA) as a protein assay test [114]. An amount of 30 μg of protein from the striatum samples was applied per lane. Protein denaturation was carried out for 5 min at 95 °C in Laemmli sample buffer containing 2% SDS, 10% glycerol, 5% mercaptoethanol, 62.5 mM Tris (pH = 6.8), and 0.004% bromophenol blue. Electrophoresis was performed in a 12% polyacrylamide gel, and the proteins were then transferred to a nitrocellulose membrane (Hybond-enhanced chemiluminescence, Amersham Biosciences, Slough, UK) overnight at 30 mA. The loading of proteins and their uniform transfer to the membrane were confirmed via Ponceau-S staining [115,116]. Ponceau-S was removed using a tris-salt buffer (TBST). The nonspecific reaction on the membrane was blocked for 1 h with TBST containing 0.05% Tween 20 (Sigma-Aldrich, Saint Louis, MO, USA) and 5% BSA at 20 °C. The membranes were then incubated with one of the first monoclonal antibodies to TH (1:1000, T1299, Sigma-Aldrich, Saint Louis, MO, USA), to TH Ser19 (1:1000, PA5-104765, Invitrogen, Walthan, MA, USA), to TH Ser40 (1:1000, NB300-173, NovusBio, Littleton, CO, USA), and to TH Ser31 (1:1000, 3370S, Cell Signalings, Danvers, MA, USA), diluted in TBST containing 1% BSA, overnight, at 4 °C. After washing in TBST, the membranes were incubated with the second antibodies conjugated with horseradish peroxidase for 2 h at 20 °C. To visualize the protein, the membranes, previously washed in TBST to remove the excess of the second antibodies, were incubated in 0.1 M Tris-HCl containing 12.5 mM luminol, 2 mM coumaric acid, and 0.03% H_2_O_2_ (pH = 8.5). The chemiluminescence intensity of the bands was recorded on a ChemiDoc Touch (Biorad, Hercules, CA, USA). The resulting chemiluminescence intensity was measured densitometrically using ImageLab software version 6.0.1 (Biorad, Hercules, CA, USA). The intensity of the bands was normalized to the total signal of the bands stained using Ponceau-S and was presented as the optical density of the bands in the experimental groups relative to the control bands, taken as 100% (Figure S1).

### 4.10. Statistical Analysis

Statistical analysis was performed using GraphPad Prism 6.0 software (GraphPad Software, San Diego, CA, USA). The compliance of the sample with normal distribution was checked using the Shapiro–Wilk test. For pairwise comparison of samples, the parametric Student’s test and the non-parametric Mann–Whitney test were used. When comparing three or more samples, a one-way analysis of variance and Tukey’s test were used for post-analysis. The data are presented as mean ± SEM. *p* ≤ 0.05 was used throughout as the significance criterion.

## Figures and Tables

**Table 1:** Changes in the gene expression of proteins involved in synaptic neurotransmission, neurodegeneration, and neuroplasticity 1, 6, and 24 h after administering 1-methyl-4-phenyl-1,2,3,6-tetrahydropyridine (MPTP) to mice twice at a single dose of 6 mg/kg (“*n*” per group = 6). The data are presented as a ratio to the level in the control, taken as 1. Statistics indicate significance with control group by using the parametric Student’s test.

Gene	Protein	Function	Time after 2 × 6 mg/kg MPTP					
			1 h		6 h		24 h	
			Fold Change	*p*	Fold Change	*p*	Fold Change	*p*
DA synthesis, degradation, transport, and reception								
*Th*	Tyrosine hydroxylase	DA synthesis	0.74	0.10	0.81	0.23	0.56	0.01
*Maoa*	Monoamine oxidase B	DA degradation	0.96	0.88	1.29	0.00	0.88	0.70
*Maob*	Monoamine oxidase B	DA degradation	0.89	0.50	0.62	0.01	0.91	0.67
*Comt*	Catechol-O-methyltranserase	DA degradation	1.21	0.02	1.15	0.17	1.20	0.20
*Slc6a3*	Dopamine transporter	DA reuptake	0.80	0.09	0.89	0.26	0.61	0.01
*Slc18a2*	Vesicular monoamine transporter 2	DA vesicles uptake	0.90	0.28	0.97	0.70	0.63	0.01
*Drd2*	Dopamine receptor 2	DA reception	0.59	0.01	0.53	0.04	0.50	0.00
Axonal transport and microtubules								
*Dynll1*	Dynein light chain	Axonal transport	0.55	0.02	0.62	0.02	1.01	0.96
*Mapt*	Microtubule-associated tau	Axonal transport	1.20	0.01	0.96	0.72	1.03	0.29
*Map2*	Microtubule-associated protein 2	Axonal transport	1.55	0.00	1.30	0.02	1.33	0.03
Vesicle cycle for neurotransmission								
*Snca*	α-Synuclein	Neurotransmission	1.11	0.42	1.05	0.74	0.77	0.03
*Syn1*	Synapsin 1	Vesicular cycle	1.59	0.01	1.34	0.08	1.28	0.15
*Syt11*	Synaptotagmin 11	Endocytosis	1.29	0.04	1.06	0.63	1.06	0.66
*Nsf*	N-ethylmaleimide sensitive fusion protein	Vesicular cycle	1.51	0.00	1.26	0.05	1.32	0.02
*Dnm1l*	Dynamin 1-like protein	Vesicular cycle Mitochondrial fission	1.34	0.02	1.25	0.05	1.21	0.06
Protein degradation								
*Ube2n*	Ubiquitin conjugating enzyme E2 N	E2 enzyme	1.07	0.41	0.85	0.04	1.05	0.69
*Psmb4*	Proteasome 20S subunit beta 4	Proteasome subunits	1.29	0.01	1.10	0.18	0.90	0.28
*Psmd4*	Proteasome 26S subunit ubiquitin receptor, non-ATPase 4	Proteasome subunits	1.20	0.05	1.48	0.00	1.31	0.23
*Usp47*	Ubiquitin specific peptidase 47	Protein deubiquitination	1.70	0.00	1.72	0.00	1.41	0.04
Neuroprotection								
*Sod1*	Superoxide dismutase 1	Antioxidant system	1.30	0.03	1.12	0.31	1.01	0.60
*Gpx1*	Glutathione peroxidase 1	Antioxidant system	1.30	0.01	1.17	0.17	1.04	0.65
*Txnrd1*	Thioredoxin reductase 1	Antioxidant system	2.59	0.01	2.32	0.01	2.41	0.01
*Prdx1*	Peroxiredoxin 1	Antioxidant system	0.73	0.03	0.94	0.53	0.75	0.34
*Nfe2l2*	Nuclear factor erythroid 2-related factor 2	Transcriptome factor	2.31	0.02	1.82	0.12	1.84	0.12
*Keap1*	Kelch-like ECH-associated protein 1	Regulation of Nfe2l2	1.49	0.01	1.48	0.00	1.04	0.78
*Ntrk2*	Neurotrophic receptor tyrosine kinase 2	Neurotrophic factor receptor	1.30	0.00	1.22	0.00	1.06	0.26
*Calb1*	Calbindin 1	Ca^2+^-binding protein	1.48	0.18	1.20	0.37	2.25	0.03
Inflammation and glial activation								
*Cnr1*	Cannabinoid receptor 1	Endocannabinoid system	1.60	0.01	1.49	0.03	1.66	0.02
*Clk1*	CDC-like kinase 1	Pre-mRNA processing	1.63	0.00	1.06	0.70	1.55	0.00

DA—dopamine, ER—endoplasmic reticulum.

**Table 2:** List of genes clustered by protein function, which are presented on chips produced by Thermo Fisher Scientific for RT-PCR with Open Array Technology.

Cluster of Genes	Genes
House-keeping gene	*Cyc1*
Dopamine synthesis, degradation, transport, and reception	*Th*, *Ddc*, *Dbh*, *Pnmt*, *Maoa*, *Maob*, *Comt*, *Slc6a3*, *Slc18a2*, *Slc29a4*, *Drd1–Drd5*
Axonal transport and microtubules	*Kif1a*, *Kif1b*, *Kif5a*, *Kif2c*, *Dync1h1*, *Dynll1*, *Dctn1*, *Mapt*, *Map2*, *Mark2*, *Tubb3*, *Tuba1a*
Vesicle cycle for neurotransmission	*Snca*, *Syn1*, *Stx1a*, *Syt1*, *Syt11*, *Rab5a*, *Rab7*, *Nsf*, *Dnm1l*
Neuroprotection	*Sod1*, *Gpx1*, *Gsr*, *Txnrd1*, *Nos1*, *Prdx1*, *Nfe2l2*, *Agtr2*, *Keap1*, *Sigmar1*, *Cacna1d*, *Trpm2*, *Bdnf*, *Gdnf*, *Ngf*, *Vegfa*, *Cdnf*, *Ntrk2*, *Ntrk1*, *Ngfr*, *Nr4a2*, *Mmp3*, *Pitx3*, *Wnt11*, *Ctnnb1*, *Calb1*
Protein degradation	*Park2*, *Ube2n*, *Uba3*, *Psmb4*, *Psmc3*, *Psmd4*, *Usp47*, *Ubb*
Inflammation and glial activation	*Gfap*, *Ifng*, *Tgfb1*, *Akt1*, *Cnr1*, *Ptgs2*, *Traf1*, *Cxcl11*
Apoptosis, necrosis, autophagy, ER stress	*Casp1*, *Casp3*, *Parp1*, *Aifm1*, *Bcl2l11*, *Map3k5*, *Cib1*, *Trp53*, *Bax*, *Fos*, *Mapk8*, *Lamp2*, *Atg16l1*, *Atg5*, *Capn1*, *Tnf*, *Ctsb*, *Ern2*, *Eif2ak3*, *Atf6*

## Data Availability

The data presented in this study are available on request from the corresponding author. The data are not publicly available due to legal issues.

## Supplementary Materials

The supporting information can be downloaded at: https://www.mdpi.com/article/10.3390/ijms25021354/s1.

## Abbreviations

3-MT	3-methoxytyramine
AADC	Aromatic L-amino acid decarboxylase
BSA	Bovine serum albumin
DA	Dopamine
DAergic	Dopaminergic
DAT	Dopamine transporter
DOPAC	3,4-dihydroxyphenylacetic acid
HPLC with ED/FD	High-performance liquid chromatography with electrochemical and fluorescence detection
HVA	Homovanillic acid
L-DOPA	L-3,4-dihydroxyphenylalaninePCR
MPP+	1-methyl-4-phenylpyridinium
MPTP	1-methyl-4-phenyl-1,2,3,6-tetrahydropyridine
NSD-1015	3-hydroxybenzylhydrazine
PBS	Phosphate-buffered saline
PD	Parkinson’s disease
PCR	Polymerase chain reaction
SN	Substantia nigra
TH	Tyrosine hydroxylase
VMAT2	Vesicular monoamine transporter 2
