# Supplementary material for: Initial Molecular Mechanisms of the Pathogenesis of Parkinson’s Disease in a Mouse Neurotoxic Model of the Earliest Preclinical Stage of This Disease
Source: Int J Mol Sci. 2024 Jan 22;25(2):1354. doi: 10.3390/ijms25021354 (PMC10816442; doi:10.3390/ijms25021354)
Supplement: Supplementary file 1 [file ijms-25-01354-s001.zip › ijms-2810453-supplementary.pdf]

Table S1. Genes and their target names on PCR chips for Open Array technology

No	Gene	Target Name	Reporter
1	*Cyc1*	Mm00470540_m1	FAM
2	*Th*	Mm00447557_m1	FAM
3	*Ddc*	Mm00516688_m1	FAM
4	*Dbh*	Mm00460472_m1	FAM
5	*Pnmt*	Mm00476993_m1	FAM
6	*Maoa*	Mm00558004_m1	FAM
7	*Maob*	Mm00555412_m1	FAM
8	*Comt*	Mm00514377_m1	FAM
9	*Slc6a3*	Mm00438388_m1	FAM
10	*Slc18a1*	Mm00461868_m1	FAM
11	*Slc18a2*	Mm00553058_m1	FAM
12	*Drd1*	Mm02620146_s1	FAM
13	*Drd2*	Mm00438545_m1	FAM
14	*Drd3*	Mm00432887_m1	FAM
15	*Drd4*	Mm00432893_m1	FAM
16	*Drd5*	Mm04210376_s1	FAM
17	*Kif1a*	Mm00492863_m1	FAM
18	*Kif1b*	Mm00801813_m1	FAM
19	*Kif5a*	Mm00515265_m1	FAM
20	*Kif2c*	Mm00728630_s1	FAM
21	*Dync1h1*	Mm00466548_m1	FAM
22	*Dynll1*	Mm00850282_g1	FAM
23	*Dctn1*	Mm01184845_m1	FAM
24	*Mapt*	Mm00521988_m1	FAM
25	*Map2*	Mm00485231_m1	FAM
26	*Mark2*	Mm01220150_g1	FAM
27	*Tubb3*	Mm00727586_s1	FAM
28	*Tuba1a*	Mm00846967_g1	FAM
29	*Snca*	Mm01188700_m1	FAM
30	*Syn1*	Mm00449772_m1	FAM
31	*Stx1a*	Mm00444008_m1	FAM
32	*Syt1*	Mm00436858_m1	FAM
33	*Syt11*	Mm00444517_m1	FAM
34	*Rab5a*	Mm00727887_s1	FAM
35	*Rab7*	Mm00784318_sH	FAM
36	*Nsf*	Mm00435390_m1	FAM
37	*Dnm1l*	Mm01342903_m1	FAM
38	*Vps35*	Mm00458167_m1	FAM
39	*Sod1*	Mm01344233_g1	FAM
40	*Gpx1*	Mm00656767_g1	FAM
41	*Gsr*	Mm00439154_m1	FAM
42	*Txnrd1*	Mm00443675_m1	FAM
43	*Nos1*	Mm01208059_m1	FAM
44	*Prdx1*	Mm01621996_s1	FAM
45	*Nfe2l2*	Mm00477784_m1	FAM
46	*Agtr2*	Mm00431727_g1	FAM
47	*Keap1*	Mm00497268_m1	FAM
48	*Sigmar1*	Mm01223547_g1	FAM
49	*Cacna1d*	Mm01209927_g1	FAM
50	*Trpm2*	Mm00663098_m1	FAM
51	*Park2*	Mm01323528_m1	FAM
52	*Ube2n*	Mm00779119_s1	FAM
53	*Uba3*	Mm00495866_m1	FAM
54	*Psmb4*	Mm01263563_m1	FAM
55	*Psmc3*	Mm00477177_m1	FAM
56	*Psmd4*	Mm01263490_m1	FAM
57	*Usp47*	Mm00659716_m1	FAM
58	*Ubb*	Mm01622233_g1	FAM
59	*Bdnf*	Mm04230607_s1	FAM
60	*Gdnf*	Mm00599849_m1	FAM
61	*Ngf*	Mm00443039_m1	FAM
62	*Vegfa*	Mm00437306_m1	FAM
63	*Cdnf*	Mm00617407_m1	FAM
64	*Ntrk2*	Mm00435422_m1	FAM
65	*Ntrk1*	Mm01219406_m1	FAM
66	*Ngfr*	Mm00446296_m1	FAM
67	*Nr4a2*	Mm00443060_m1	FAM
68	*Mmp3*	Mm00440295_m1	FAM
69	*Pitx3*	Mm01194166_g1	FAM
70	*Wnt11*	Mm00437327_g1	FAM
71	*Ctnnb1*	Mm00483039_m1	FAM
72	*Slc29a4*	Mm00525575_m1	FAM
73	*Calb1*	Mm00486647_m1	FAM
74	*Ifng*	Mm01168134_m1	FAM
75	*Tgfb1*	Mm01178820_m1	FAM
76	*Akt1*	Mm01331626_m1	FAM
77	*Cnr1*	Mm01212171_s1	FAM
78	*Ptgs2*	Mm00478374_m1	FAM
79	*Clk1*	Mm00438254_m1	FAM
80	*Traf1*	Mm00493827_m1	FAM
81	*Cxcl11*	Mm00444662_m1	FAM
82	*Casp1*	Mm00438023_m1	FAM
83	*Casp3*	Mm01195085_m1	FAM
84	*Parp1*	Mm01321084_m1	FAM
85	*Aifm1*	Mm00442548_m1	FAM
86	*Bcl2l11*	Mm00437796_m1	FAM
87	*Map3k5*	Mm00434883_m1	FAM
88	*Cib1*	Mm00501944_m1	FAM
89	*Trp53*	Mm01731290_g1	FAM
90	*Bax*	Mm00432051_m1	FAM
91	*Fos*	Mm00487425_m1	FAM

92	*Mapk8*	Mm00489514_m1	FAM
93	*Lamp2*	Mm00495267_m1	FAM
94	*Atg16l1*	Mm00513085_m1	FAM
95	*Atg5*	Mm01187303_m1	FAM
96	*Capn1*	Mm00482964_m1	FAM
97	*Tnf*	Mm00443258_m1	FAM
98	*Ctsb*	Mm01310506_m1	FAM
99	*Ern2*	Mm00469005_m1	FAM
100	*Eif2ak3*	Mm00438700_m1	FAM
101	*Atf6*	Mm01295319_m1	FAM
102	*Gfap*	Mm01253033_m1	FAM

Table S2. The main characteristics of the substantia nigra and striatum 1, 3, 6, and 24 h after twice administration of MPTP at a single dose of 6 mg/kg.

Time after NaCl or MPTP injections	1 h		3 h		6 h		24 h	
Substance	Control	MPTP	Control	MPTP	Control	MPTP	Control	MPTP
Substantia nigra								
DA, pmol	4.07 ± 0.57	1.89 ± 0.33	4.81 ± 0.31	1.94 ± 0.22	4.46 ± 0.25	2.51 ± 0.16	4.65 ± 0.28	4.27 ± 0.50
DOPAC, pmol	1.81 ± 0.20	0.19 ± 0.01	1.93 ± 0.15	0.22 ± 0.01	2.16 ± 0.12	0.27 ± 0.02	1.94 ± 0.20	2.22 ± 0.13
HVA, pmol	2.00 ± 0.24	1.32 ± 0.18	2.34 ± 0.11	1.21 ± 0.03	2.03 ± 0.08	1.21 ± 0.09	1.94 ± 0.14	2.21 ± 0.15
TH activity, % of control	100 ± 5.3	72.8 ± 6.8	100 ± 5.3	64.5 ± 6.1	100 ± 5.3	77.1 ± 6.0	100 ± 6.0	93.0 ± 8.0
DOPAC/DA, % of control	100 ± 10.6	20.7 ± 8.3	100 ± 10.1	24.5 ± 16.1	100 ± 11.8	21.5 ± 7.0	100 ± 9.7	88.2 ± 5.2
HVA/DA, % of control	100 ± 10.9	143.9 ± 15.1	100 ± 3.0	129.2 ± 8.9	100 ± 2.7	110.0 ± 4.2	100 ± 5.8	123.5 ± 6.2
Striatum								
Number of varicose swellings of nerve fibers immunopositive for TH, AADC and DAT, (units)	985.6 ± 31.2	904.3 ± 23.2	933.8 ± 12.5	798.7 ± 41.8	1089.0 ± 51.3	873.3 ± 55.7	1057.2 ± 50.7	757.5 ± 51.1
Area of nerve fibers immunopositive for TH, AADC and DAT, μm^2	970.0 ± 47.8	861.9 ± 62.6	870.3 ± 26.9,	710.9 ± 42.0	1178.6 ± 39.3	869 ± 111.2	1172.9 ± 381.	690.6 ± 75.7
DA, pmol/mg	103.1 ± 0.9	104.2 ± 2.4	103.9 ± 1.2	75.3 ± 3.0	103.5 ± 1.3	69.2 ± 3.9	104.7 ± 1.5	54.9 ± 3.8
DOPAC, pmol/mg	7.29 ± 0.30	1.56 ± 0.07	7.48 ± 0.20	2.76 ± 0.29	7.15 ± 0.23	2.12 ± 0.15	7.35 ± 0.38	4.96 ± 0.51
3-MT, pmol/mg	1.28 ± 0.14	1.42 ± 0.11	1.21 ± 0.11	3.81 ± 0.66	1.37 ± 0.16	0.98 ± 0.05	1.59 ± 0.04	1.60 ± 0.08
HVA, pmol/mg	9.88 ± 0.46	6.44 ± 0.25	10.73 ± 0.31	8.09 ± 0.50	9.80 ± 0.45	5.50 ± 0.15	9.59 ± 0.43	6.44 ± 0.25
TH activity	100.0 ± 5.0	178.6 ± 5.0	100.0 ± 5.0	32.6 ± 4.8	100.0 ± 5.0	83.7 ± 5.1	100.0 ± 1.7	67.1 ± 3.8
DOPAC/DA, % of control	100 ± 3.2	20.8 ± 5.2	100 ± 2.5	56.1 ± 13.5	100 ± 2.4	49.3 ± 9.1	100 ± 6.9	116.2 ± 5.9
HVA/DA, % of control	100 ± 4.2	63.0 ± 6.7	100 ± 1.7	115.0 ± 8.7	100 ± 3.1	88.4 ± 7.6	100 ± 3.4	108.0 ± 7.7
3-MT/DA, % of control	100 ± 7.7	110.5 ± 9.9	100 ± 9.3	433.8 ± 18.6	100 ± 12.4	115.1 ± 14.6	100 ± 3.8	165.2 ± 6.8
TH content, % of control	100 ± 4.3	98.9 ± 5.9	100 ± 6.2	89.5 ± 6.2	100 ± 4.0	96.7 ± 5.5	100 ± 4.7	82.3 ± 4.0
TH-P19 content, % of control	100 ± 1.9	76.4 ± 8.2	100 ± 7.3	60.0 ± 5.6	100 ± 9.1	92.2 ± 4.4	100 ± 0.9	94.8 ± 6.5
TH-P31 content, % of control	100 ± 8.8	104.4 ± 9.0	100 ± 8.7	68.5 ± 3.8	100 ± 7.1	78.6 ± 8.0	100 ± 5.9	81.4 ± 6.8
TH-P40 content, % of control	100 ± 10.6	120.5 ± 3.5	100 ± 4.0	90.0 ± 1.4	100 ± 4.5	84.2 ± 4.2	100 ± 10.0	88.7 ± 10.4

3-MT – 3-methoxytyramine; AADC – aromatic L-amino acid decarboxylase; DA – dopamine; DAT – dopamine transporter; DOPAC – 3,4-dihydroxyphenylacetic acid; HVA – homovanillic acid; TH – tyrosine hydroxylase; TH-P19 – tyrosine hydroxylase phosphorylated at Ser19; TH-P31 – tyrosine hydroxylase phosphorylated at Ser31; TH-P40 – tyrosine hydroxylase phosphorylated at Ser40.

Table S3. Changes in the gene expression of proteins involved in synaptic neurotransmission, neurodegeneration, and neuroplasticity 1, 6, 24 h after administering of 1-methyl-4-phenyl-1,2,3,6-tetrahydropyridine (MPTP) to mice twice at a single dose of 6 mg/kg ("*n*" per group = 6). The data are presented as a ratio to the level in the control, taken as 1. Statistics indicate significance with control group by parametric Student's test.

Gene	Protein	Function	Time after 2x6 mg/kg MPTP					
			1 h		6 h		24 h	
			Fold change	p	Fold change	p	Fold change	p
**DA synthesis, degradation, transport and reception**								
Th	Tyrosine hydroxylase	DA synthesis	0.74	0.10	0.81	0.23	0.56	0.01
Ddc	Dopa decarboxylase	DA synthesis	1.37	0.07	1.28	0.10	0.77	0.24
Maoa	Monoamine oxidase B	DA degradation	0.96	0.88	1.29	0.00	0.88	0.70
Maob	Monoamine oxidase B	DA degradation	0.89	0.50	0.62	0.01	0.91	0.67
Comt	Catechol-O-methyltranserase	DA degradation	1.21	0.02	1.15	0.17	1.20	0.20
Slc6a3	Dopamine transporter	DA reuptake	0.80	0.09	0.89	0.26	0.61	0.01
Slc18a2	Vesicular monoamine transporter 2	DA vesicles uptake	0.90	0.28	0.97	0.70	0.63	0.01
Drd2	Dopamine receptor 2	DA reception	0.59	0.01	0.53	0.04	0.50	0.00
**Axonal transport and microtubules**								
Kif1a	Kinesin	Axonal transport	1.17	0.08	1.14	0.15	1.09	0.22
Kif5a	Kinesin	Axonal transport	0.85	0.95	0.76	0.60	0.95	0.61
Dynll1	Dynein light chain	Axonal transport	0.55	0.02	0.62	0.02	1.01	0.96
Dctn1	Dynactin 1	Axonal transport	1.22	0.38	1.24	0.25	1.08	0.64
Mapt	Microtubule-associated tau	Axonal transport	1.20	0.01	0.96	0.72	1.03	0.29
Map2	Microtubule-associated protein 2	Axonal transport	1.55	0.00	1.30	0.02	1.33	0.03
Tubb3	β-tubulin	Axonal transport	0.99	0.96	0.90	0.22	0.84	0.58
Tuba1a	α-tubulin	Axonal transport	1.07	0.69	0.87	0.25	0.97	0.61
**Vesicle cycle for neurosmission**								
Snca	α-Synuclein	Neurotransmission	1.11	0.42	1.05	0.74	0.77	0.03
Syn1	Synapsin 1	Vesicular cycle	1.59	0.01	1.34	0.08	1.28	0.15
Syt1	Synaptotagmin 1	Vesicular cycle	1.02	0.85	1.08	0.38	0.92	0.51
Syt11	Synaptotagmin 11	Endocytosis	1.29	0.04	1.06	0.63	1.06	0.66
Rab5a	RAB5A	Endocytosis	1.46	0.08	1.14	0.66	1.28	0.22
Rab7	RAB7	Endocytosis	1.08	0.54	1.05	0.71	1.09	0.47
Nsf	N-ethylmaleimide sensitive fusion protein	Vesicular cycle	1.51	0.00	1.26	0.05	1.32	0.02
Dnm1l	Dynamin 1-like protein	Mitochondrial fission	1.34	0.02	1.25	0.05	1.21	0.06
Vps35	Vacuolar protein sorting ortholog 35	Vesicular cycle	1.41	0.01	1.21	0.07	1.49	0.00
**Protein degradation**								
Ube2n	Ubiquitin Conjugating Enzyme E2 N	E2 enzyme	1.07	0.41	0.85	0.04	1.05	0.69
Uba3	Ubiquitin like modifier activating enzyme 3	E1 enzyme	1.34	0.11	1.11	0.56	1.33	0.06

Psmb4	Proteasome 20S Subunit Beta 4	Proteasome subunits	1.29	0.01	1.10	0.18	0.90	0.28
Psmc3	Proteasome 26S Subunit. ATPase 3	Proteasome subunits	1.07	0.86	0.97	0.73	1.02	0.94
Psmd4	Proteasome 26S subunit ubiquitin receptor. non-ATPase 4	Proteasome subunits	1.20	0.05	1.48	0.00	1.31	0.23
Usp47	Ubiquitin specific peptidase 47	Protein deubiquitination	1.70	0.00	1.72	0.00	1.41	0.04
Ubb	Ubiquitin B	Targeting cellular proteins for degradation	1.19	0.14	1.11	0.27	1.12	0.27
Ctsb	Cystatin-B	Protein degradation	1.07	0.51	0.93	0.82	1.08	0.33
**Neuroprotection**								
Sod1	Superoxide dismutase 1	Antioxidant system	1.30	0.03	1.12	0.31	1.01	0.60
Gpx1	Glutathione peroxidase 1	Antioxidant system	1.30	0.01	1.17	0.17	1.04	0.65
Gsr	Glutathione reductase	Antioxidant system	1.36	0.76	1.62	0.82	0.86	0.71
Txnrd1	Thioredoxin reductase 1	Antioxidant system	2.59	0.01	2.32	0.01	2.41	0.01
Prdx1	Peroxiredoxin 1	Antioxidant system	0.73	0.03	0.94	0.53	0.75	0.34
Nfe2l2	Nuclear factor erythroid 2-related factor 2	Transcriptome factor (regulation of antioxidant system)	2.31	0.02	1.82	0.12	1.84	0.12
Keap1	kelch-like ECH-associated protein 1	Regulation of Nfe2l2	1.49	0.01	1.48	0.00	1.04	0.78
Sigmar1	Sigma-1 receptor	Chaperone protein, Calcium signaling	1.02	0.83	1.10	0.57	0.99	0.97
Ntrk2	Neurotrophic receptor tyrosine kinase 2	Neurotrophic factor receptor	1.30	0.00	1.22	0.00	1.06	0.26
Nr4a2	Nuclear receptor subfamily 4 group A member 2	Transcriptome factor	1.10	0.96	0.80	0.32	0.49	0.05
Calb1	Calbindin 1	Ca^{2+}-binding protein	1.48	0.18	1.20	0.37	2.25	0.03
**Inflammation and glial activation**								
Gfap	Glial fibrillary acidic protein	Glial activation	0.64	0.20	0.79	0.28	0.56	0.74
Akt1	Protein kinase B alpha	Cell proliferation. survival	1.11	0.51	1.07	0.66	0.91	0.85
Cnr1	Cannabinoid Receptor 1	Pre-mRNA processing	1.60	0.01	1.49	0.03	1.66	0.02
Clk1	CDC Like Kinase 1	Endocannabinoid system	1.63	0.00	1.06	0.70	1.55	0.00
**Apoptosis, necrosis, autophagy, ER stress**								
Parp1	Poly [ADP-ribose] polymerase 1	DNA reparation, apoptosis	1.18	0.28	0.96	0.99	0.98	0.62
Cib1	Ca^{2+} and integrin binding 1	Apoptosis	1.60	0.06	1.01	0.79	1.01	0.79
Aifm1	Apoptosis inducing factor mitochondria associated 1	Apoptosis	1.34	0.28	1.10	0.65	1.29	0.44

Bax	Bax protein	Apoptosis	1.10	0.47	1.24	0.08	0.97	0.83
Mapk8	Mitogen-activated protein kinase 8	Apoptosis	1.32	0.24	0.91	0.41	1.30	0.23
Lamp2	Lysosomal associated membrane protein 2	Autophagy	1.16	0.11	1.01	0.80	1.06	0.49
Atg5	Autophagy related 5	Autophagy	1.64	0.09	1.26	0.53	1.36	0.40
Trp53	Transformation related protein 53	Reulation apoptosis, DNA repair	0.86	0.96	1.12	0.45	0.84	0.40

DA—dopamine, ER – endoplasmic reticulum.

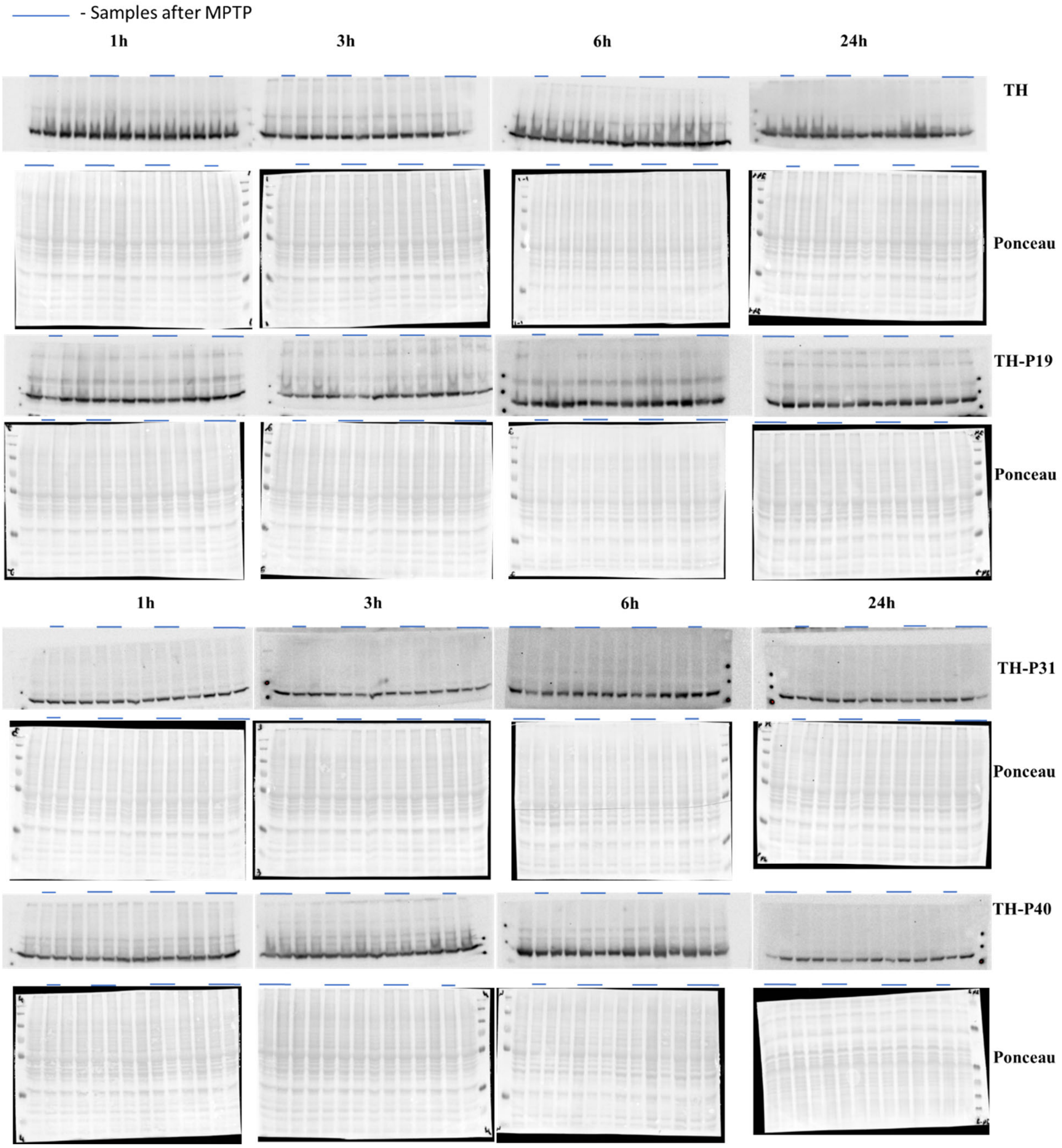

Figure S1. Representative western blot of tyrosine hydroxylase (TH), its phosphorylated forms at Ser19, Ser31, and Ser40 and membranes stained by Ponceau 1, 3, 6, and 24 h after twice administration of 1-methyl-4-phenyl-1,2,3,6-tetrahydropyridine (MPTP) at a dose of 6 mg/kg and in the control.
